# Global Assessment of COVID-19 Mortality Displacement From 2020 to 2024

**DOI:** 10.1001/jamanetworkopen.2025.55442

**Published:** 2026-01-29

**Authors:** Xiyin Chen, Edward Ye, Benjamin John Cowling, David Makram Bishai

**Affiliations:** 1 School of Public Health, Li Ka Shing Faculty of Medicine, The University of Hong Kong, Hong Kong SAR, China

## Abstract

**Importance:**

The COVID-19 pandemic disrupted long-term mortality declines. Preliminary 2024 data indicate a return to prepandemic trends, but part of this reduction may reflect mortality displacement, in which frail or high-risk individuals died earlier than they otherwise would have during the pandemic, possibly leading to temporarily fewer deaths in subsequent years.

**Objective:**

To estimate global all-cause mortality trends during and after COVID-19 and assess the association of mortality displacement with mortality declines seen in 2023 and 2024.

**Design, Setting, and Participants:**

This retrospective cross-national, cross-sectional time-series study assessed mortality data from January 2015 to December 2024, with January 2015 to December 2019 serving as the prepandemic baseline for country-specific, age-adjusted linear projections of expected mortality. Countries were included with high-quality national mortality reporting from the Human Mortality Database Short-Term Mortality Fluctuations. Total populations for each country were stratified by sex and age (0-14 years, 15-64 years, 65-74 years, 75-84 years, and ≥85 years).

**Exposures:**

The COVID-19 pandemic years (2020-2022 or 2020-2023) and postpandemic years (2023-2024 or 2024 alone), relative to a 2015-to-2019 prepandemic baseline.

**Main Outcomes and Measures:**

Excess mortality is defined as actual minus expected mortality, in which expected deaths were projected from prepandemic (2015-2019) data using an overdispersed Poisson regression model. Mortality displacement was defined as a statistically significant postpandemic mortality deficit in 2023 and 2024 (or in 2024) following a positive excess mortality during the 2020-2022 (or 2020-2023) pandemic period. To quantify the magnitude of mortality displacement, the mortality displacement ratio was defined as the ratio of the cumulative postpandemic deficit to the cumulative pandemic-period excess.

**Results:**

This study included 352 182 284 deaths across 34 countries from 2015 to 2024, with a median (IQR) 2020 population composition of 50.75% (50.33%-51.44%) female and 19.64% (17.74%-20.64%) aged 65 years or older. Statistically significant mortality displacement was detected in 3 of 34 countries (Greece: 10% [95% CI, 4%-15%], Latvia: 21% [95% CI, 14%-28%], and Poland: 21% [95% CI, 17%-25%]), predominantly among adults aged 85 years or older and in strata in the highest quintile of COVID-19–era excess mortality. In 2024, the US returned to its prepandemic pattern of stable mortality, with an excess mortality rate of 3 (95% CI, −2 to 7) per 100 000. In contrast, most European countries had not yet resumed their prepandemic trajectories, exhibiting excess mortality rates ranging from 11 (95% CI, 3-18) per 100 000 in France to 115 (95% CI, 94-135) per 100 000 in Lithuania.

**Conclusions and Relevance:**

In this cross-sectional study of 34 countries, postpandemic mortality declines were rarely associated with mortality displacement; most European countries had not returned to their prepandemic mortality trajectories by 2024. These results suggest that pandemic strategies should prioritize population-wide protection and highlight the need to understand why so many European countries have failed to resume their pre–COVID-19 pattern of mortality decline.

## Key Points

**Question:**

Did mortality declines observed in 2023 and 2024 represent a return to prepandemic trends, or were they associated with a temporary drop in deaths among frail individuals who died earlier than expected during the COVID-19 pandemic (mortality displacement)?

**Findings:**

In this cross-sectional study of 352 182 284 deaths across 34 countries, statistically significant mortality displacement were observed in only 3 countries (Greece, Latvia, and Poland), mainly among adults aged 85 years or older and in countries with high COVID-19–era mortality. The US resumed stable prepandemic mortality patterns by 2024, but most European countries had not.

**Meaning:**

These findings underscore the need for broad population-wide protection during pandemics and for understanding why many countries have yet to return to pre–COVID-19 mortality trajectories.

## Teaser

This cross-sectional study of 34 countries estimates global all-cause mortality trends during and after the COVID-19 pandemic and assesses whether mortality declines in 2023 and 2024 could represent mortality displacement.

## Introduction

For most of the last century, high-income countries experienced annual declines in age-specific mortality rates.^1^ However, the COVID-19 pandemic created upturns in mortality rates between 2020 and 2021 in many high-income countries.^2,3,4^ In the wake of the pandemic, some mortality rates have resumed their pre–COVID-19 pattern of annual declines. However, a portion of the mortality decline observed immediately after a pandemic can be explained by mortality displacement (also referred to as the mortality harvesting effect, harvesting, and the harvesting effect). This occurs when the pandemic causes frail or high-risk individuals, who would have died in the near future, to die earlier than they otherwise would have. As a result, the months or years following the crisis may show fewer deaths than expected because many of the most at-risk individuals had already died prematurely.^5^ This demographic phenomenon has been documented after previous health crises, including epidemics, pandemics, severe influenza seasons, and heat waves, and could therefore be plausibly observed following COVID-19 as well.^6,7,8^

In parallel, other factors may drive postpandemic mortality decline. Rapid scale-up of vaccination campaigns reduced COVID-19–related mortality and helped prevent future waves.^9,10^ On average, easing COVID-19 mobility and cross-border restrictions coincided with a rebound in trade and travel, contributing to a partial economic recovery and some improvements in employment and poverty, which are key social determinants of health, although these gains were highly unequal between and within countries.^11,12,13,14,15,16,17^ Together, these multiple processes shape observed mortality trends after a pandemic and underscore the need to distinguish the temporary mortality displacement from longer-term recovery. Disentangling the contribution of mortality displacement from other population health drivers is crucial to accurately assess the pandemic’s true burden and to guide public health strategies during system recovery.^18^

There are various methods to calculate excess mortality.^19,20,21,22,23^ Overall, these studies affirm the general approach to estimate excess deaths relative to a projected baseline of expected deaths before the pandemic. While commonly used during periods of elevated mortality, the method also applies when observed deaths fall below baseline, often termed negative excess or deficit mortality. In both cases, estimates are benchmarked against the counterfactual of uninterrupted prepandemic trends. However, a critical gap involves quantifying the extent of the mortality displacement on mortality across different age groups immediately following the COVID-19 pandemic. Prior studies focusing on excess mortality have a limited scope, concentrating on the pandemic period and not covering the postpandemic recovery phase.

Bor et al^22^ provided insights into postpandemic mortality declines in the US, and Riou et al^24^ provided findings of those in Switzerland; however, a multicountry analysis of the mortality displacement across age groups, to our knowledge, is largely absent. Thus, this study systematically measured the mortality displacement using high-quality data available by country, year, age, and sex following the COVID-19 pandemic.

## Methods

### Data Source

In this cross-sectional study, we used the Short-Term Mortality Fluctuations dataset, which compiles harmonized weekly all-cause mortality statistics from national vital registration systems of multiple countries. We extracted weekly mortality data covering the period from January 2015 through December 2024 for 34 selected countries. Most included countries were classified as high-income economies by the World Bank; Bulgaria (newly reclassified to high-income in fiscal year 2025) was included because of its reliable and harmonized mortality surveillance system.^25^ This study represents a secondary analysis of publicly available aggregate data from the Human Mortality Database, without patient or public involvement; as such, this study did not require institutional review board approval or informed patient consent, in accordance with 45 CFR §46. The study followed the Strengthening the Reporting of Observational Studies in Epidemiology (STROBE) reporting guideline.

Mortality data were stratified by country, year, week, sex, and age groups (0-14 years, 15-64 years, 65-74 years, 75-84 years, and ≥85 years). The Short-Term Mortality Fluctuations dataset provided both weekly death counts and weekly death rates, with death rates calculated as the number of deaths occurring each week divided by the annual population exposure, normalized to weekly units. Data for each year typically included 52 weeks; week 53 was excluded to ensure comparability across years. Countries included in the analysis were categorized into 5 quintiles based on the severity of their cumulative excess mortality from January 2020 to December 2022. Mortality displacement was hypothesized to have been highest in countries with the highest January 2020 to December 2022 excess mortality and negligible in countries with the lowest January 2020 to December 2022 mortality. Quintiles were defined from the lowest excess mortality (quintile 1) to the highest excess mortality (quintile 5), providing a structured comparison of pandemic outcomes across countries.

### Statistical Analysis

We defined 2020 to 2022 (or 2020 to 2023) as the pandemic period, and 2023 to 2024 (or only 2024) as the postpandemic period; this definition was applied consistently in all analyses. To illustrate the analytic concept, a stylized mortality index for 2015 to 2025 under 2 counterfactual scenarios, 1 with mortality displacement (Figure, A) and 1 without (Figure, B), is shown. In both panels, the prepandemic trend from 2015 to 2019 is extrapolated and represents the number of deaths expected had the pandemic not occurred. A temporary spike in deaths from 2020 to 2022 is shown in the Figure. In the scenario without mortality displacement, deaths returned to the projected trend from 2023 onward; in the scenario with mortality displacement, deaths fell below the projected trend in 2023 and 2024, creating a visible gap in the prepandemic trend.

**Figure.**
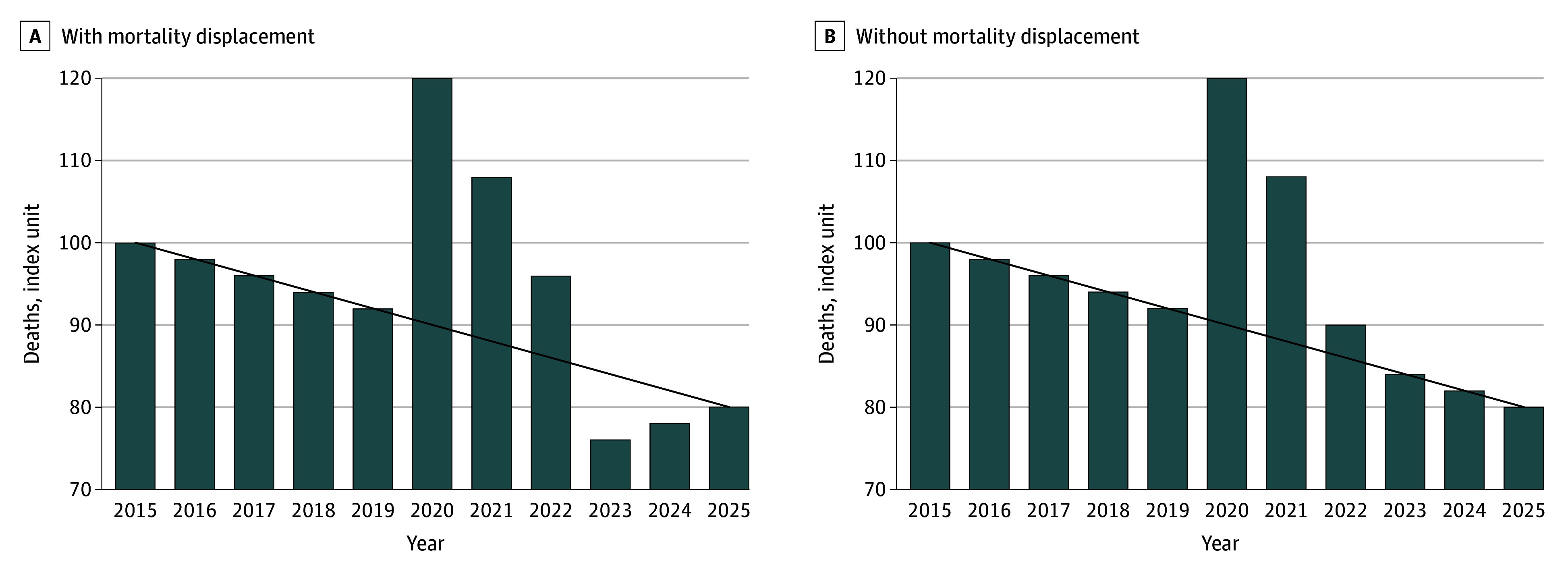
Stylized Mortality Index Under 2 Counterfactual Scenarios, 2015 to 2025 The black lines extrapolate the prepandemic trend from 2015 to 2019 and represent the number of deaths expected had the pandemic not occurred. In the mortality displacement scenario, the deficit below the line indicates mortality displacement.

In our empirical analysis, we treated this gap as an association with mortality displacement and quantified it in 3 steps. First, for each country and year, we project the expected number of deaths in 2020 to 2024 by extending the 2015-to-2019 trend. Second, we summed the excess deaths from 2020 to 2022 (or from 2020 to 2023), defined as deaths above this expected level. Third, we summed the mortality deficit during 2023 to 2024 (or only 2024), defined as deaths falling below the expected level. The mortality displacement fraction was then interpreted as the share of the initial excess deaths in 2020 to 2022 (or 2020 to 2023) that was paid back by the mortality deficit in 2023 to 2024 or only 2024 (ie, the ratio of the postpandemic deficit to the pandemic-period excess). The methodologic details for each step are described.

The expected weekly deaths were estimated with an overdispersed Poisson generalized linear model using historical mortality data from the prepandemic period (2015-2019). For each country (*c*), sex (*s*), and age group (*a*), we fitted prepandemic data (week 1, 2015, to week 52, 2019) as follows in Equation 1^19,21,26^: 
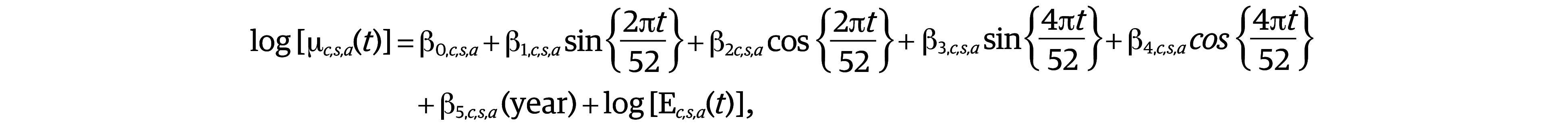
in which *μ_c,s,a_*(*t*) is the expected number of deaths at week *t*; *E_c,s,a_*(*t*) is the weekly person-weeks exposure used as an offset; and *β_5,c,s,a_*(year) adjusted for temporal trend. Two harmonic pairs captured the dominant annual and semiannual seasonal components. Robust SEs were used to accommodate overdispersion. Based on each country’s parameters for age and sex, we projected death counts for week 1, 2020, to week 52, 2024. Afterward, we compared the observed weekly number of deaths in 2020 to 2024 with the expected number of weekly deaths for each country, stratified by age and sex, to estimate weekly excess mortality. Specifically, weekly excess deaths for 2020 to 2024 were calculated in Equation 2: excess*_c_*_,_*_s_*_,_*_a_*(*t*) = *d_c,s,a_*(*t*) − *μ̂_c,s,a_*(*t*), in which *μ̂_c,s,a_*(*t*) is the fitted value from Equation 1. Annual excess mortality rates per 100 000 *R_c,s,a,y_* were obtained by Equation 3:



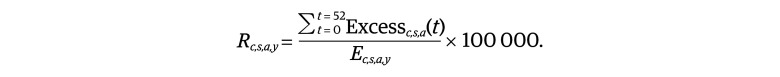



Given the linear trend in expected mortality derived from 2015 to 2019, having an excess mortality rate of 0 in 2023 or 2024 would imply exactly resuming the established pre–COVID-19 mortality decline. If a country did better than achieving 0 excess mortality in 2023 or 2024, then it signals the potential presence of mortality displacement. It is also possible that a country superseded its pre–COVID-19 population health performance by improving the overall health system. However, if the country was in a high quintile for COVID-19–era excess deaths, then mortality displacement became a more plausible explanation. To isolate mortality displacement from chance, we defined mortality displacement as a sustained cumulative postpandemic mortality deficit in 2023 and 2024 or in 2024, conditional on a positive excess in 2020 to 2022 (or 2020 to 2023). Deficits were assessed against the bootstrapped 95% prediction interval of the prepandemic baseline. For each qualifying stratum, we then computed the cumulative excess mortality during the initial surge (2020-2022 or 2020-2023) and the subsequent deficit (2023-2024 or 2024). The mortality displacement fraction^20^ was finally expressed in Equation 4:
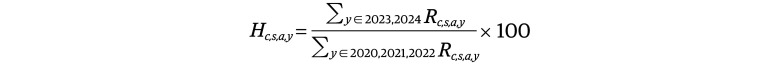
or Equation 5:



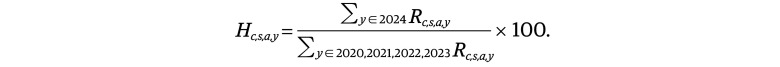



To obtain 95% CIs for both *R_c,s,a,y_*, we performed nonparametric bootstrapping with 1000 resamples. For each bootstrap replicate, we resampled the weekly mortality data with replacement within each country-sex-age stratum. For each resample, we reestimated the expected deaths by refitting Equation 1, recomputing excess deaths and the excess death rate in Equations 2 and 3, and recalculating the mortality displacement ratio in Equations 4 and 5. The 2.5 percentile of the resulting bootstrap distributions was taken as the lower 95% CI bound and the 97.5 percentile as the upper 95% CI bound.

We conducted 3 prespecified sensitivity analyses to evaluate robustness: (1) refitting the baseline model using a negative binomial specification, (2) fitting a Poisson generalized estimating equation with an autoregressive (AR[1]) working correlation structure to account for serial correlation in weekly deaths, and (3) repeating all models using broader age bands (0-64 years, 65-74 years, 75-84 years, and ≥85 years) to reduce sparsity in age-specific strata. To address the risk of an inflated type I error from multiple hypothesis testing, we used the Benjamini-Hochberg adjustment to control the false discovery rate (*q* ≤ 0.05). We compared the main results with the unadjusted *P* values to assess the outcome of the adjustment. A 2-sided *P* < .05 was considered statistically significant. All calculations were performed using Stata, version 18.5 (StataCorp LLC).

## Results

Our analysis included 352 182 284 deaths in 34 countries from 2015 to 2024; the median (IQR) population composition in 2020 was 50.75% (50.33%-51.44%) females, 49.25% (48.56%-49.67%) males, and 19.64% (17.74%-20.64%) aged 65 years or older. Based on Equation 1, we found that in the prepandemic period (2015-2019), 13 countries (Australia, Iceland, Israel, Luxembourg, Norway, New Zealand, Canada, Switzerland, Sweden, Belgium, Spain, England and Wales, and Austria) exhibited significant negative mortality trends (eg, Australia: rate, −0.0212 [95% CI, −0.0234 to −0.0190]; *P *<* *.001), 8 (Portugal, Slovenia, Czechia, Greece, Bulgaria, Croatia, Latvia, and Poland) showed significant positive trends (eg, Portugal: rate, 0.0096 [95% CI, 0.0033 to 0.0160]; *P* = .003), and the remaining 13 were fairly similar over time (eTable 3 in Supplement 1). We defined baselines as well-estimated if they were significant.

The cumulative excess death rate per 100 000 (based on mean population) for 2020 to 2022 compared with expected deaths based on 2015-to-2019 mortality is provided in Table 1 and for 2020 to 2024 in Table 2. Positive values indicate more deaths than expected; negative values indicate fewer deaths than expected. The 34 countries were grouped into 5 quintiles according to their cumulative excess death rate in 2020 to 2022 (CEDR20-22). In the lowest quintile, the CEDR20-22 values were −21 (95% CI, −35 to −6) per 100 000 for New Zealand, 3 (95% CI, −37 to 42) per 100 000 for Luxembourg, 26 (95% CI, 6 to 46) per 100 000 for Denmark, 81 (95% CI, 68 to 94) per 100 000 for Australia, 82 (95% CI, 62 to 103) per 100 000 for Norway, 108 (95% CI, 93 to 124) for per 100 000 Israel, and 113 (95% CI, 62 to 165) per 100 000 for Iceland, with all values except Luxembourg achieving statistical significance. New Zealand’s value of −21 indicates that it was able to sustain better-than-expected mortality declines even during the 2020-to-2022 period. New Zealand’s reduction persisted through 2020 to 2024 (−35), and Denmark (−21) and Luxembourg (−100) also recorded reductions. New Zealand pulled off statistically significantly negative cumulative excess mortality from 2020 to 2024 in all age groups except ages 75 to 84 years. Notably, Denmark and Luxembourg, with the lowest COVID-19–era mortality, exhibited significant cumulative negative excess mortality among individuals aged 85 years or older from 2020 to 2024. The remaining quintile 1 countries, namely Denmark, Australia, Norway, Israel, and Iceland, failed to sustain negative values and showed statistically significant excess mortality from 2020 to 2024. Conversely, CEDR20-22 values for Poland were 520 (95% CI, 457 to 584) per 100 000; Latvia, 540 (95% CI, 474 to 606) per 100 000; Slovakia, 549 (95% CI, 487 to 611) per 100 000; Croatia, 600 (95% CI, 532 to 668) per 100 000; Lithuania, 814 (95% CI, 740 to 887) per 100 000; and Bulgaria, 1070 (95% CI, 955 to 1186) per 100 000.

**Table 1.** Cumulative Excess Death Rate Per 100 000 Population From 2020 to 2022 by Country Compared With Expected Deaths Based on 2015 to 2019 Mortality

Excess mortality quintile^a^	Country	Cumulative excess deaths per 100 000 (95% CI)^b^
1	New Zealand	−21 (−35 to −6)
1	Luxembourg	3 (−37 to 42)
1	Denmark	26 (6 to 46)
1	Australia	81 (68 to 94)
1	Norway	82 (62 to 103)
1	Israel	108 (93 to 124)
1	Iceland	113 (62 to 165)
2	Sweden	131 (105 to 156)
2	Canada	142 (128 to 157)
2	Finland	167 (140 to 194)
2	France	201 (171 to 231)
2	Germany	212 (180 to 244)
2	Switzerland	213 (178 to 249)
2	Netherlands	230 (199 to 261)
3	Northern Ireland	235 (196 to 273)
3	Belgium	258 (212 to 304)
3	England and Wales	268 (226 to 311)
3	Portugal	271 (225 to 318)
3	Scotland	280 (239 to 321)
3	Spain	301 (251 to 350)
3	Slovenia	319 (265 to 373)
4	Austria	321 (283 to 359)
4	Greece	355 (315 to 395)
4	Estonia	358 (313 to 403)
4	United States	405 (375 to 434)
4	Italy	443 (393 to 493)
4	Hungary	456 (398 to 514)
4	Czechia	514 (451 to 577)
5	Poland	520 (457 to 584)
5	Latvia	540 (474 to 606)
5	Slovakia	549 (487 to 611)
5	Croatia	600 (532 to 668)
5	Lithuania	814 (740 to 887)
5	Bulgaria	1070 (955 to 1186)

^a^ Quintile 1 had the lowest excess mortality and quintile 5 had the highest.

^b^ Based on mean population. Example interpretation: The excess death rate of 1070 means that between 2020 and 2022, there were 1070 cumulative deaths per 100 000 residents on average compared with the number expected if prepandemic trends had continued.

**Table 2.** Cumulative Excess Death Rate Per 100 000 Population From 2020 to 2024 by Country Compared With Expected Deaths Based on 2015 to 2019 Mortality

Excess mortality quintile^a^	Country	Cumulative excess deaths per 100 000 (95% CI)^b^
1	Luxembourg	−100 (−150 to −49)
1	New Zealand	−35 (−53 to −16)
1	Denmark	−21 (−47 to 6)
1	Israel	126 (105 to 146)
1	Norway	152 (128 to 177)
1	Australia	158 (142 to 175)
2	Sweden	169 (140 to 199)
2	Canada	172 (155 to 189)
2	France	225 (194 to 257)
2	Iceland	233 (168 to 299)
2	Finland	259 (225 to 293)
2	Germany	261 (224 to 298)
2	Portugal	297 (247 to 348)
2	Switzerland	301 (262 to 340)
3	Belgium	306 (257 to 354)
3	Greece	323 (278 to 367)
3	Slovenia	330 (273 to 388)
3	Netherlands	341 (307 to 375)
3	Estonia	364 (310 to 419)
3	New England and Wales	366 (319 to 412)
3	Spain	386 (332 to 439)
4	Northern Ireland	394 (344 to 443)
4	Scotland	404 (353 to 455)
4	Poland	413 (351 to 475)
4	Latvia	429 (358 to 501)
4	United States	431 (401 to 462)
4	Austria	446 (401 to 490)
4	Hungary	474 (412 to 535)
5	Italy	529 (477 to 581)
5	Slovakia	549 (487 to 610)
5	Czechia	578 (513 to 642)
5	Croatia	703 (630 to 776)
5	Lithuania	1028 (951 to 1105)
5	Bulgaria	1127 (1009 to 1245)

^a^ Quintile 1 had the lowest excess mortality and quintile 5 had the highest.

^b^ Based on mean population. Example interpretation: The excess death rate of 1127 means that between 2020 and 2024 there were 1127 cumulative deaths per 100 000 residents on average compared with the number expected if prepandemic trends had continued.

The cumulative excess deaths per 100 000 population from 2020 to 2024 by country and age group, relative to expected deaths based on 2015-to-2019 mortality is shown in Table 3. Notably, 22 of 34 countries recorded negative or insignificantly positive cumulative excess mortality from 2020 to 2024 for those aged 0 to 14 years. However, only 6 countries recorded reduced excess mortality for individuals aged older than 14 years, among which 4 of 6 countries were in quintile 1, and the remaining 2 countries had statistically insignificant reductions. In all countries, older age groups recorded much greater cumulative excess mortality compared with younger age groups across the entire 2020-to-2024 period.

**Table 3.** Cumulative Excess Deaths Per 100 000 Population From 2020 to 2024 by Country and Age Group Relative to Expected Deaths Based on 2015 to 2019 Mortality

Excess mortality quintile^a^	Country	Cumulative excess deaths per 100 000 (95% CI)^b^				
		Ages 0-14 y	Ages 15-64 y	Ages 65-74 y	Ages 75-84 y	Ages ≥85 y
1	Australia	0 (−1 to 2)	31 (26 to 37)	217 (179 to 254)	1040 (923 to 1157)	2813 (2320 to 3306)
1	Denmark	9 (0 to 17)	10 (−0 to 21)	−155 (−233 to −77)	563 (372 to 754)	−2388 (−3173 to −1604)
1	Iceland	90 (61 to 119)	−39 (−75 to −3)	1247 (969 to 1525)	326 (−381 to 1033)	6541 (4121 to 8962)
1	Israel	4 (−3 to 11)	55 (30 to 80)	470 (397 to 544)	343 (142 to 543)	2991 (2290 to 3692)
1	Luxembourg	7 (−22 to 36)	−108 (−136 to −80)	71 (−166 to 309)	−147 (−670 to 376)	−1268 (−2859 to 323)
1	Norway	−4 (−10 to 3)	32 (23 to 42)	98 (21 to 175)	840 (636 to 1043)	3214 (2425 to 4002)
1	New Zealand	−20 (−23 to −18)	−26 (−38 to −15)	−5 (−68 to 58)	404 (247 to 560)	−1907 (−2653 to −1161)
2	Canada	10 (8 to 12)	79 (70 to 87)	345 (304 to 385)	1216 (1107 to 1324)	533 (3 to 1064)
2	Switzerland	15 (8 to 22)	37 (30 to 45)	156 (86 to 225)	1468 (1254 to 1682)	5900 (4845 to 6955)
2	Germany	−0 (−2 to 2)	82 (74 to 91)	680 (613 to 748)	1763 (1581 to 1946)	−273 (−1149 to 602)
2	Finland	−5 (−11 to 1)	39 (26 to 51)	340 (264 to 416)	991 (801 to 1180)	4081 (3370 to 4792)
2	France	−2 (−4 to 1)	2 (−5 to 8)	175 (124 to 226)	658 (488 to 827)	4726 (4072 to 5380)
2	Netherlands	−3 (−7 to 2)	67 (60 to 74)	506 (441 to 571)	1945 (1736 to 2154)	4814 (3826 to 5803)
2	Sweden	17 (12 to 22)	47 (39 to 54)	50 (−18 to 118)	1291 (1116 to 1467)	1342 (545 to 2139)
3	Belgium	−36 (−41 to −31)	65 (54 to 76)	293 (196 to 390)	1398 (1091 to 1705)	5170 (3918 to 6422)
3	Spain	4 (1 to 7)	23 (15 to 31)	394 (308 to 480)	1518 (1213 to 1823)	6900 (5708 to 8092)
3	England and Wales	9 (6 to 12)	96 (83 to 110)	491 (378 to 605)	1611 (1286 to 1936)	5946 (4648 to 7243)
3	Northern Ireland	3 (−10 to 15)	88 (59 to 118)	1164 (997 to 1331)	2190 (1796 to 2584)	4459 (3081 to 5836)
3	Scotland	11 (3 to 19)	31 (11 to 52)	1012 (886 to 1139)	1177 (844 to 1510)	7916 (6797 to 9035)
3	Portugal	−20 (−26 to −13)	27 (15 to 40)	441 (357 to 524)	659 (388 to 931)	5032 (4039 to 6025)
3	Slovenia	−5 (−16 to 5)	65 (43 to 88)	528 (373 to 683)	2295 (1870 to 2719)	2573 (1111 to 4035)
4	Austria	−4 (−10 to 2)	82 (71 to 93)	506 (421 to 591)	3869 (3624 to 4114)	2734 (1769 to 3698)
4	Czechia	−22 (−27 to −17)	92 (71 to 114)	903 (690 to 1117)	3852 (3392 to 4312)	8083 (6531 to 9634)
4	Estonia	23 (8 to 38)	271 (241 to 302)	553 (344 to 762)	1615 (1228 to 2001)	631 (−561 to 1823)
4	Greece	0 (−6 to 6)	71 (55 to 87)	530 (432 to 628)	1494 (1263 to 1725)	2605 (1713 to 3497)
4	Hungary	15 (8 to 22)	97 (62 to 131)	1301 (1075 to 1526)	2828 (2308 to 3348)	3235 (1913 to 4556)
4	Italy	−4 (−6 to −1)	72 (64 to 81)	679 (588 to 770)	2095 (1849 to 2342)	5944 (5107 to 6781)
4	United States	17 (15 to 19)	195 (172 to 218)	981 (882 to 1079)	2537 (2317 to 2757)	3738 (3059 to 4418)
5	Bulgaria	−8 (−18 to 2)	238 (176 to 299)	2384 (1940 to 2829)	5497 (4658 to 6335)	14 405 (12 421 to 16 388)
5	Croatia	4 (−6 to 15)	58 (33 to 83)	1699 (1509 to 1889)	3462 (2954 to 3969)	8573 (7207 to 9940)
5	Lithuania	15 (3 to 26)	442 (405 to 480)	2070 (1858 to 2282)	4281 (3807 to 4755)	8077 (6652 to 9502)
5	Latvia	2 (−11 to 15)	204 (156 to 251)	1246 (1001 to 1490)	2454 (1929 to 2979)	−647 (−2191 to 896)
5	Poland	−15 (−18 to −12)	34 (5 to 63)	996 (766 to 1226)	2124 (1567 to 2680)	7695 (6287 to 9104)
5	Slovakia	−4 (−14 to 7)	142 (109 to 176)	1380 (1123 to 1637)	4258 (3603 to 4914)	5038 (3551 to 6525)

^a^ Quintile 1 had the lowest excess mortality and quintile 5 had the highest.

^b^ Based on mean population. Example interpretation: An excess of 1380 in the 65-to-74–year age group means 1380 cumulative deaths per 100 000 people aged 65 to 74 years from 2020 to 2024 compared with what would have been expected from prepandemic trends.

The annual excess mortality per 100 000 population and the estimated mortality displacement by country from 2020 to 2024 are provided in Table 4. Even though in quintile 1, the highest observed mortality displacement ratios were found in Denmark at 180% and in Luxembourg at 2770%, these results were statistically insignificant and likely reflect their relatively low early excess mortality combined with substantial subsequent mortality deficits, rather than a large absolute displacement of deaths. However, 3 countries exhibited significant mortality displacement. In particular, Greece at 10% (95% CI, 4%-15%), Latvia at 21% (95% CI, 14%-28%), and Poland at 21% (95% CI, 17%-25%) showed mortality displacement ratios with statistical significance. By 2024, the US had returned to its prepandemic stable trend (3 [95% CI, −2 to 7] per 100 000). In contrast, most European countries (including Norway, France, Switzerland, the Netherlands, Belgium, Spain, the UK, Austria, Italy, and Lithuania) had not yet resumed their prepandemic trajectories, exhibiting excess mortality rates ranging from 11 (95% CI, 3 to 18) per 100 000 in France to 115 (95% CI, 94 to 135) per 100 000 in Lithuania.

**Table 4.** Annual Excess Death Rate Per 100 000 Population and Estimated Mortality Displacement by Country From 2020 to 2024 Relative to Expected Deaths Based on 2015 to 2019 Mortality

Excess mortality quintile^a^	Country	Annual excess deaths per 100 000 (95% CI)					Mortality displacement ratio (95% CI), %^b^
		2020	2021	2022	2023	2024	
1	Australia	−16 (−21 to −11)	15 (10 to 20)	81 (67 to 94)	38 (31 to 46)	39 (32 to 46)	NA
1	Denmark	−21 (−31 to −10)	12 (−2 to 25)	35 (23 to 46)	−2 (−13 to 9)	−44 (−57 to −30)	180 (−217 to 577)
1	Iceland	3 (−25 to 31)	9 (−18 to 37)	99 (64 to 133)	58 (29 to 87)	60 (28 to 92)	NA
1	Israel	28 (19 to 36)	42 (34 to 50)	39 (29 to 48)	5 (−10 to 20)	14 (9 to 19)	NA
1	Luxembourg	25 (1 to 49)	1 (−20 to 22)	−22 (−43 to −2)	−45 (−67 to −24)	−54 (−75 to −33)	2770 (−41 884 to 47 424)
1	Norway	−9 (−18 to −0)	14 (1 to 27)	77 (62 to 92)	35 (24 to 45)	35 (24 to 46)	NA
1	New Zealand	−45 (−56 to −35)	−16 (−23 to −10)	40 (30 to 50)	8 (−0 to 17)	−22 (−29 to −15)	NA
2	Canada	33 (25 to 42)	32 (26 to 38)	76 (65 to 87)	33 (27 to 39)	−3 (−11 to 5)	2 (−2 to 5)
2	Switzerland	94 (62 to 125)	41 (28 to 54)	79 (62 to 95)	46 (34 to 58)	43 (32 to 53)	NA
2	Germany	28 (12 to 43)	71 (53 to 89)	113 (91 to 136)	49 (36 to 62)	−0 (−14 to 14)	0 (−3 to 3)
2	Finland	6 (−4 to 16)	36 (23 to 49)	125 (104 to 146)	80 (61 to 100)	12 (1 to 23)	NA
2	France	73 (51 to 96)	57 (44 to 69)	71 (55 to 88)	14 (6 to 22)	11 (3 to 18)	NA
2	Netherlands	76 (54 to 98)	87 (67 to 106)	68 (54 to 81)	55 (45 to 65)	57 (48 to 66)	NA
2	Sweden	73 (54 to 92)	18 (7 to 29)	40 (28 to 51)	35 (23 to 46)	4 (−5 to 14)	NA
3	Belgium	153 (109 to 196)	36 (23 to 49)	70 (54 to 86)	24 (14 to 34)	25 (15 to 34)	NA
3	Spain	147 (99 to 194)	63 (49 to 77)	91 (71 to 111)	37 (28 to 46)	49 (34 to 64)	NA
3	England and Wales	119 (85 to 154)	85 (64 to 107)	64 (48 to 79)	66 (51 to 81)	32 (22 to 42)	NA
3	Northern Ireland	85 (61 to 109)	86 (62 to 111)	64 (44 to 83)	66 (42 to 89)	93 (72 to 115)	NA
3	Scotland	103 (73 to 133)	99 (80 to 118)	78 (59 to 97)	81 (60 to 101)	44 (28 to 59)	NA
3	Portugal	87 (65 to 108)	96 (62 to 131)	89 (67 to 110)	20 (8 to 32)	8 (−8 to 23)	NA
3	Slovenia	144 (96 to 192)	107 (81 to 134)	68 (48 to 88)	14 (−4 to 31)	−2 (−19 to 14)	1 (−3 to 4)
4	Austria	91 (67 to 116)	103 (84 to 123)	126 (104 to 148)	79 (62 to 95)	47 (31 to 64)	NA
4	Czechia	151 (113 to 189)	269 (226 to 312)	94 (75 to 113)	30 (18 to 43)	32 (20 to 45)	NA
4	Estonia	18 (−3 to 40)	221 (187 to 255)	119 (95 to 143)	11 (−13 to 35)	−5 (−28 to 18)	1 (−4 to 7)
4	Greece	56 (40 to 73)	171 (139 to 203)	129 (105 to 152)	−1 (−12 to 11)	−34 (−51 to −17)	10 (4 to 15)
4	Hungary	100 (65 to 135)	271 (228 to 313)	85 (69 to 102)	10 (−2 to 22)	5 (−10 to 21)	NA
4	Italy	174 (135 to 213)	127 (108 to 147)	141 (115 to 167)	54 (43 to 66)	30 (18 to 42)	NA
4	United States	141 (120 to 162)	166 (148 to 185)	98 (84 to 111)	25 (21 to 30)	3 (−2 to 7)	NA
5	Bulgaria	220 (158 to 281)	624 (536 to 711)	227 (182 to 271)	9 (−5 to 22)	37 (18 to 55)	NA
5	Croatia	128 (90 to 166)	300 (249 to 350)	173 (142 to 203)	45 (29 to 61)	56 (37 to 75)	NA
5	Lithuania	193 (152 to 234)	386 (337 to 436)	235 (200 to 270)	97 (78 to 116)	115 (94 to 135)	NA
5	Latvia	38 (12 to 64)	353 (302 to 405)	150 (122 to 178)	−5 (−30 to 20)	−109 (−139 to −79)	21 (14 to 28)
5	Poland	159 (117 to 201)	278 (234 to 322)	83 (67 to 99)	−39 (−48 to −30)	−71 (−81 to −62)	21 (17 to 25)
5	Slovakia	86 (63 to 109)	356 (303 to 410)	106 (89 to 124)	5 (−7 to 17)	−5 (−16 to 7)	1 (−1 to 3)

Abbreviation: NA, not applicable.

^a^ Quintile 1 had the lowest excess mortality and quintile 5 had the highest.

^b^ The mortality displacement ratio was defined as the proportion of cumulative excess mortality from 2020 to 2022 or from 2020 to 2023 that was offset by cumulative negative excess mortality during 2023 and 2024 combined or 2024 alone. A mortality displacement percentage exceeding 100% indicates that the subsequent mortality deficit entirely offset or surpassed the initial mortality surge; NA indicates that the required pattern of all-positive surge years followed by all-negative deficit years was not observed in that group, and therefore no mortality displacement was computed. Example interpretation: A mortality displacement of 10% indicates that 10% of the excess deaths occurring from 2020 to 2022 were later offset by lower-than-expected mortality in 2023 and 2024.

The annual excess death rate and mortality displacement by country and age group are presented in eFigures 1-5 and eTable 1 in Supplement 1. Significant mortality displacement were observed in 0 of 3 countries in the 0-to-14–year age group, in 6 of 15 countries in the 15-to-64–year age group, in 5 of 15 countries in the 65-to-74–year age group, and in 4 of 8 countries in the 75-to-84–year age group, with effect magnitudes ranging from 7% to 68% in the 15-to-64–year age group, 8% to 62% in the 65-to-74–year age group, and 9% to 42% in the 75-to-84–year age group. Notably, in the 85 years or older age group, the mortality displacement was more pronounced, with 10 of 13 countries showing significant mortality displacement, ranging from 6% to 106%. Across all 25 significant age-group findings, 40% were from countries in mortality-burdened quintile 5, and 32% were from countries in mortality-burdened quintile 4, indicating that the highest burdened countries were disproportionately associated with the observed significant mortality displacement. The mortality displacement did not differ significantly between females and males in the younger age groups, as shown in eTable 2 in Supplement 1. However, in the 85 years or older age group, the difference was more pronounced, with 12 of 34 countries exhibiting insignificantly higher mortality displacement in females compared with only 3 countries where mortality displacement was higher in males.

The sensitivity analyses generally supported the robustness of our findings (eTables 4-6 in Supplement 1). For Greece, Latvia, and Poland, mortality displacement ratios and 95% CIs were very similar across the negative binomial, the AR(1) generalized estimating equation, and alternative age-band specifications, and all 3 countries remained classified as having significant mortality displacement. In the US, however, the 2024 excess mortality estimate was close to 0 and not statistically different from the baseline in the primary model (3 [95% CI, −2 to 7] per 100 000) but became a modest deficit under the AR(1) generalized estimating equation specification (−9 [95% CI, −14 to −3] per 100 000), consistent with a possible mortality displacement. Because these estimates are small in magnitude and lie near the null, we interpreted the US mortality displacement signal as sensitive to modeling assumptions about serial correlation. After applying the false discovery rate correction, the pattern of statistical significance generally remained consistent with the unadjusted *P* values (eTable 7 in Supplement 1). This suggests that controlling for multiple testing was not associated with the interpretation of our results.

## Discussion

This cross-sectional study found the magnitude and heterogeneity of the recovery and sustained decline in mortality across 34 countries following the COVID-19 pandemic, with results stratified by age, sex, and mortality quintile. The primary outcome of this research assesses how excess mortality during the pandemic was associated with subsequent mortality displacement. Statistically significant mortality displacement was seen in only 3 countries where pandemic-era excess mortality was high (Greece, Latvia, and Poland). They were predominantly observed among the oldest age group (≥85 years).

Our study also found the pace of recovery of pre–COVID-19 mortality trends. By 2024, the US had returned to its prepandemic pattern of relatively stable all-cause mortality. In contrast, as of 2024, most European countries, including Norway, France, Switzerland, the Netherlands, Belgium, Spain, the UK, Austria, Italy, and Lithuania, had still not resumed their prepandemic mortality trajectories. Among the 8 countries (Portugal, Slovenia, Czechia, Greece, Bulgaria, Croatia, Latvia, and Poland) with prepandemic positive mortality trends, 5 countries (Portugal, Slovenia, Czechia, Bulgaria, and Croatia) continued to experience significant excess mortality or sustained elevated baseline mortality. Of the 8 countries that had rising pre–COVID-19 mortality trends, only those achieving negative excess mortality would represent a return to conditions that were normal for high-income health systems. This situation prompts consideration of whether persistent outcomes from the pandemic, associated with sustained health system strain or depleted population health capital, may have continued to influence mortality patterns through 2024.

Our study also offers insight into the cumulative outcome of COVID-19 when including both the pandemic period of 2020 to 2022 and the recovery years of 2023 to 2024. Luxembourg uniquely demonstrated a statistically significantly faster-than-expected reduction in mortality, whereas New Zealand and Denmark maintained their expected rates of mortality decline. New Zealand had statistically significantly negative cumulative excess mortality from 2020 to 2024 in all age groups except ages 75 to 84 years. However, this was exceptional. For the 7 countries in the best quintile of low COVID-19–era mortality, most age groups experienced positive cumulative excess mortality when summing 2020 to 2024. Notably, Denmark and Luxembourg, with the lowest COVID-19–era mortality, exhibited significant cumulative negative excess mortality among individuals aged 85 years or older from 2020 to 2024. This is hard to explain and may be potentially due to factors such as smaller sample sizes, imprecise denominators, high mobility among older populations, and the suppression of influenza during 2020.

Most top-performing countries regarding overall excess mortality did not succeed in sustaining overall declines in mortality from 2020 to 2024. The worse the cumulative death rate was in 2020 to 2022, the worse the cumulative mortality was for 2020 to 2024. In other words, mortality displacement did not compensate for failing to protect the population during the pandemic. Although younger cohorts showed lower net mortality, associated with the 50-year trend toward better health of younger ages, the significant and persistent excess mortality among older adults, even in the presence of mortality displacement, demonstrates that targeted protection alone was insufficient. The scale of mortality displacement observed in the most affected countries may further indicate that any shielding strategies used in those countries failed to adequately protect high-risk groups.

Multiple factors contribute to the COVID-19 mortality displacement. Extensive studies have found that the pandemic disproportionately affected older adults and individuals with preexisting comorbidities, including type 2 diabetes,^27,28,29^ high blood pressure,^30,31,32^ and obesity.^33,34,35^ Additionally, in countries with high COVID-19 mortality rates, individuals at elevated risk might either succumb to or recover from the virus earlier, thus reducing subsequent expected mortality rates.^24^

Our findings have several policy implications. First, many European countries as of 2024 have yet to resume the typical pattern of mortality decline that characterized the last century of progress. The resumption of a postpandemic mortality decline in Greece, Latvia, and Poland plausibly may be driven primarily by the displaced timing of deaths of at-risk individuals, especially older adults in countries with high mortality, and may reflect a return to prepandemic trajectories rather than substantive improvements beyond the 2015 to 2019 downtrend. Pandemic management policies must prioritize protecting high-risk populations through comprehensive, population-wide measures, as targeted shielding alone was insufficient. In parallel, integrating retrospective analyses of age-specific excess mortality into routine surveillance frameworks may help detect potential mortality displacement. While real-time monitoring may be limited by data lags, periodic assessment of cumulative excess deaths across age groups, especially among older adults, may inform evaluations of recovery and health system resilience. Finally, future research should aim to disentangle temporary mortality displacement from genuine recovery, ensuring that public health strategies are based on an accurate understanding of a pandemic’s impact and ongoing vulnerabilities.

### Strengths and Limitations

Few studies, to our knowledge, have explicitly quantified mortality displacement after COVID-19 across such a large set of countries and demographic groups, making this a unique contribution to understanding postpandemic dynamics. A key strength of this study is its comprehensive inclusion of 34 countries with high-quality data, stratified by age, sex, and mortality quintile, which enabled the detection of heterogeneous patterns that would be masked in aggregate analyses. The use of nationally reported data, subjected to rigorous quality checks, with standardized formats, longitudinal and cross-country comparability, and detailed survival data at the oldest ages further enhanced the study’s external validity and generalizability.

This study has several limitations. First, despite using harmonized and validated national data, reporting quality and completeness may vary across countries, potentially introducing bias. Second, our analysis was ecological and based on aggregated age-group and country-level data, which limits our ability to adjust for individual-level factors such as comorbidities, socioeconomic status, or vaccination status, and raises the possibility of an ecologic fallacy; that is, inferences made about individuals from group-level data may not hold at the individual level. Third, demographic dynamics during and after the pandemic, such as selective migration of older adults, shifts in the age distribution, or differential survival of frail individuals, may vary across countries and age groups and could generate patterns that resemble mortality displacement even in the absence of true mortality displacement. Fourthly, while our study quantifies the association of mortality displacement with postpandemic mortality declines and underscores the importance of considering the mortality displacement when interpreting mortality trends after 2020, it does not fully disentangle this temporary outcome from longer-term recovery associated with other factors such as vaccination, health care improvements, and socioeconomic recovery. In addition, the observational nature of the study precludes causal inference, and findings should be interpreted in light of these constraints.

## Conclusions

The findings of this cross-sectional study suggest that 31 of 34 countries with high-quality mortality data exhibited no statistically significant evidence of mortality displacement. In contrast, for Greece, Latvia, and Poland, countries with above-average COVID-19 mortality, part of the postpandemic decline may reflect mortality displacement, particularly among the oldest age groups. However, the scale of early excess mortality far exceeded what displacement alone could make up for COVID-19. This underscores a widely seen failure of shielding strategies to adequately protect broadly defined individuals. Recognizing this distinction is essential for accurately interpreting postpandemic mortality trends and for designing policies that effectively safeguard broader populations without propagating the myth that older individuals who died in the COVID-19 era were already near death. Future research should further disentangle mortality displacement from genuine recovery to establish a clearer causal understanding of the factors driving postpandemic mortality trends, as well as unravel why so many European countries have failed to resume their pre–COVID-19 pattern of mortality decline.

## Notes

Supplement 1. **eFigure 1.** Excess Death Rate by Country, Year, and by Age Group for Quintile 1 **eFigure 2.** Excess Death Rate by Country, Year, and by Age Group for Quintile 2 **eFigure 3.** Excess Death Rate by Country, Year, and by Age Group for Quintile 3 **eFigure 4.** Excess Death Rate by Country, Year, and by Age Group for Quintile 4 **eFigure 5.** Excess Death Rate by Country, Year, and by Age Group for Quintile 5 **eTable 1.** Annual Excess Death Rate per 100,000 Population and Estimated Harvesting Effect, by Country and Age Category, 2020-2024, Relative to Expected Deaths Based on 2015-2019 Mortality **eTable 2.** Annual Excess Death Rate per 100,000 Population and Estimated Harvesting Effect, by Country, Age Group, and Sex, 2020-2024, Relative to Expected Deaths Based on 2015-2019 Mortality **eTable 3.** The Linear Trend in Mortality Rates During 2015 to 2019 by Quintile and Country **eTable 4.** Annual Excess Death Rate per 100,000 Population and Estimated Harvesting Effect, by Country, 2020-2024, Relative to Expected Deaths Based on 2015-2019 Mortality (the Sensitivity Analysis Results for the Negative Binomial Model) **eTable 5.** Annual Excess Death Rate per 100,000 Population and Estimated Harvesting Effect, by Country, 2020-2024, Relative to Expected Deaths Based on 2015-2019 Mortality (the Sensitivity Analysis Results for the Broader Age Band) **eTable 6.** Annual Excess Death Rate per 100,000 Population and Estimated Harvesting Effect, by Country, 2020-2024, Relative to Expected Deaths Based on 2015-2019 Mortality (the Sensitivity Analysis Results From the Autocorrelation Control) **eTable 7. **Annual Excess Death Rate per 100,000 Population and Estimated Harvesting Effect, by Country, 2020-2024, Relative to Expected Deaths Based on 2015-2019 Mortality (the Benjamini-Hochberg FDR Adjustment)

Supplement 2.
Data Sharing Statement
