# Supplementary material for: Global Assessment of COVID-19 Mortality Displacement From 2020 to 2024
Source: JAMA Netw Open. 2026 Jan 29;9(1):e2555442. doi: 10.1001/jamanetworkopen.2025.55442 (PMC12856679; doi:10.1001/jamanetworkopen.2025.55442)
Supplement: Supplement 1. — eFigure 1. Excess Death Rate by Country, Year, and by Age Group for Quintile 1 eFigure 2. Excess Death Rate by Country, Year, and by Age Group for Quintile 2 eFigure 3. Excess Death Rate by Country, Year, and by Age Group for Quintile 3 eFigure 4. Excess Death Rate by Country, Year, and by Age Group for Quintile 4 eFigure 5. Excess Death Rate by Country, Year, and by Age Group for Quintile 5 eTable 1. Annual Excess Death Rate per 100,000 Population and Estimated Harvesting Effect, by Country and Age Category, 2020-2024, Relative to Expected Deaths Based on 2015-2019 Mortality eTable 2. Annual Excess Death Rate per 100,000 Population and Estimated Harvesting Effect, by Country, Age Group, and Sex, 2020-2024, Relative to Expected Deaths Based on 2015-2019 Mortality eTable 3. The Linear Trend in Mortality Rates During 2015 to 2019 by Quintile and Country eTable 4. Annual Excess Death Rate per 100,000 Population and Estimated Harvesting Effect, by Country, 2020-2024, Relative to Expected Deaths Based on 2015-2019 Mortality (the Sensitivity Analysis Results for the Negative Binomial Model) eTable 5. Annual Excess Death Rate per 100,000 Population and Estimated Harvesting Effect, by Country, 2020-2024, Relative to Expected Deaths Based on 2015-2019 Mortality (the Sensitivity Analysis Results for the Broader Age Band) eTable 6. Annual Excess Death Rate per 100,000 Population and Estimated Harvesting Effect, by Country, 2020-2024, Relative to Expected Deaths Based on 2015-2019 Mortality (the Sensitivity Analysis Results From the Autocorrelation Control) eTable 7. Annual Excess Death Rate per 100,000 Population and Estimated Harvesting Effect, by Country, 2020-2024, Relative to Expected Deaths Based on 2015-2019 Mortality (the Benjamini-Hochberg FDR Adjustment) [file jamanetwopen-e2555442-s001.pdf]

Supplemental Online Content

Chen X, Ye E, Cowling BJ, Bishai DM. Global assessment of COVID-19 mortality displacement from 2020 to 2024. *JAMA Netw Open*. 2026;9(1):e2555442. doi:10.1001/jamanetworkopen.2025.55442

eFigure 1. Excess Death Rate by Country, Year, and by Age Group for Quintile 1
eFigure 2. Excess Death Rate by Country, Year, and by Age Group for Quintile 2
eFigure 3. Excess Death Rate by Country, Year, and by Age Group for Quintile 3
eFigure 4. Excess Death Rate by Country, Year, and by Age Group for Quintile 4
eFigure 5. Excess Death Rate by Country, Year, and by Age Group for Quintile 5
eTable 1. Annual Excess Death Rate per 100,000 Population and Estimated Harvesting Effect, by Country and Age Category, 2020-2024, Relative to Expected Deaths Based on 2015-2019 Mortality
eTable 2. Annual Excess Death Rate per 100,000 Population and Estimated Harvesting Effect, by Country, Age Group, and Sex, 2020-2024, Relative to Expected Deaths Based on 2015-2019 Mortality
eTable 3. The Linear Trend in Mortality Rates During 2015 to 2019 by Quintile and Country
eTable 4. Annual Excess Death Rate per 100,000 Population and Estimated Harvesting Effect, by Country, 2020-2024, Relative to Expected Deaths Based on 2015-2019 Mortality (the Sensitivity Analysis Results for the Negative Binomial Model)
eTable 5. Annual Excess Death Rate per 100,000 Population and Estimated Harvesting Effect, by Country, 2020-2024, Relative to Expected Deaths Based on 2015-2019 Mortality (the Sensitivity Analysis Results for the Broader Age Band)
eTable 6. Annual Excess Death Rate per 100,000 Population and Estimated Harvesting Effect, by Country, 2020-2024, Relative to Expected Deaths Based on 2015-2019 Mortality (the Sensitivity Analysis Results From the Autocorrelation Control)
eTable 7. Annual Excess Death Rate per 100,000 Population and Estimated Harvesting Effect, by Country, 2020-2024, Relative to Expected Deaths Based on 2015-2019 Mortality (the Benjamini-Hochberg FDR Adjustment)

This supplemental material has been provided by the authors to give readers additional information about their work.

© 2026 Chen X et al. *JAMA Network Open*.

eFigure 1. Excess Death Rate by Country, Year, and by Age Group for Quintile 1

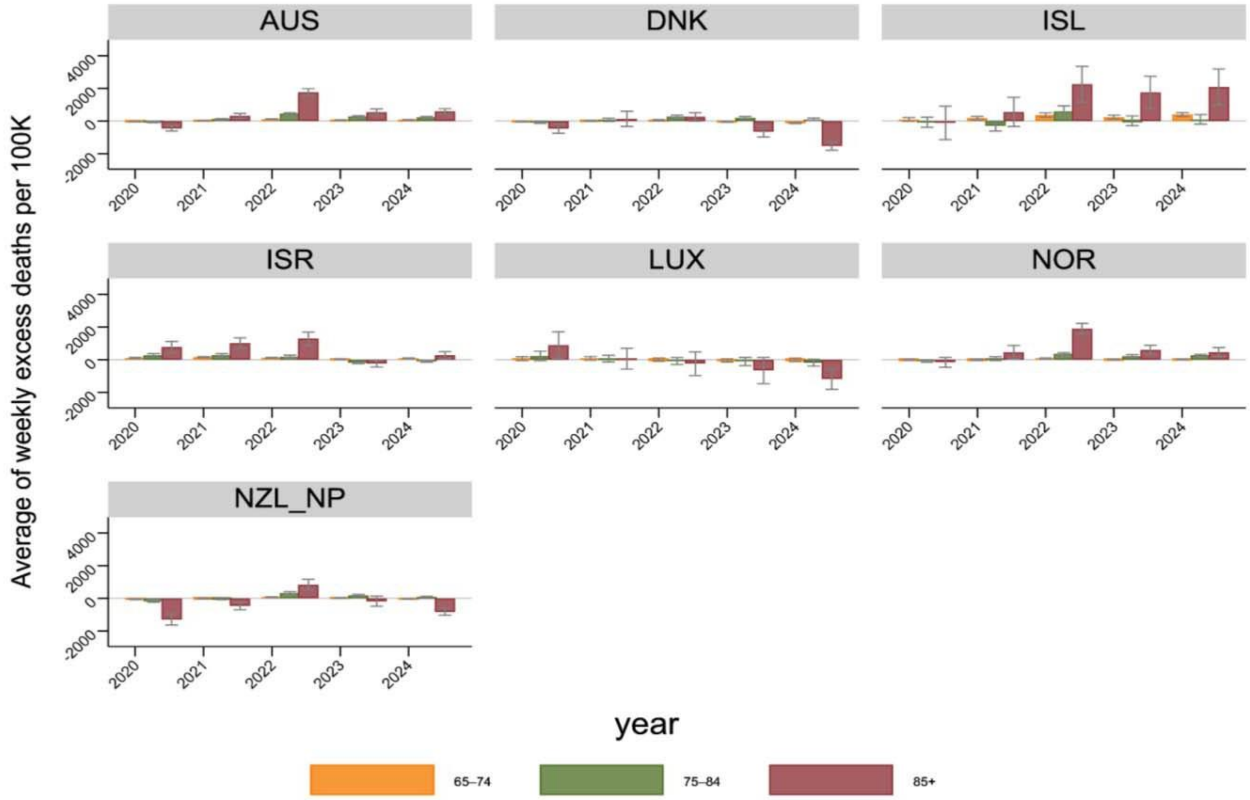

Note: AUS, Australia; DNK, Denmark; ISL, Iceland; ISR, Israel; LUX, Luxembourg; NOR, Norway; NZL_NP, New Zealand

eFigure 2. Excess Death Rate by Country, Year, and by Age Group for Quintile 2

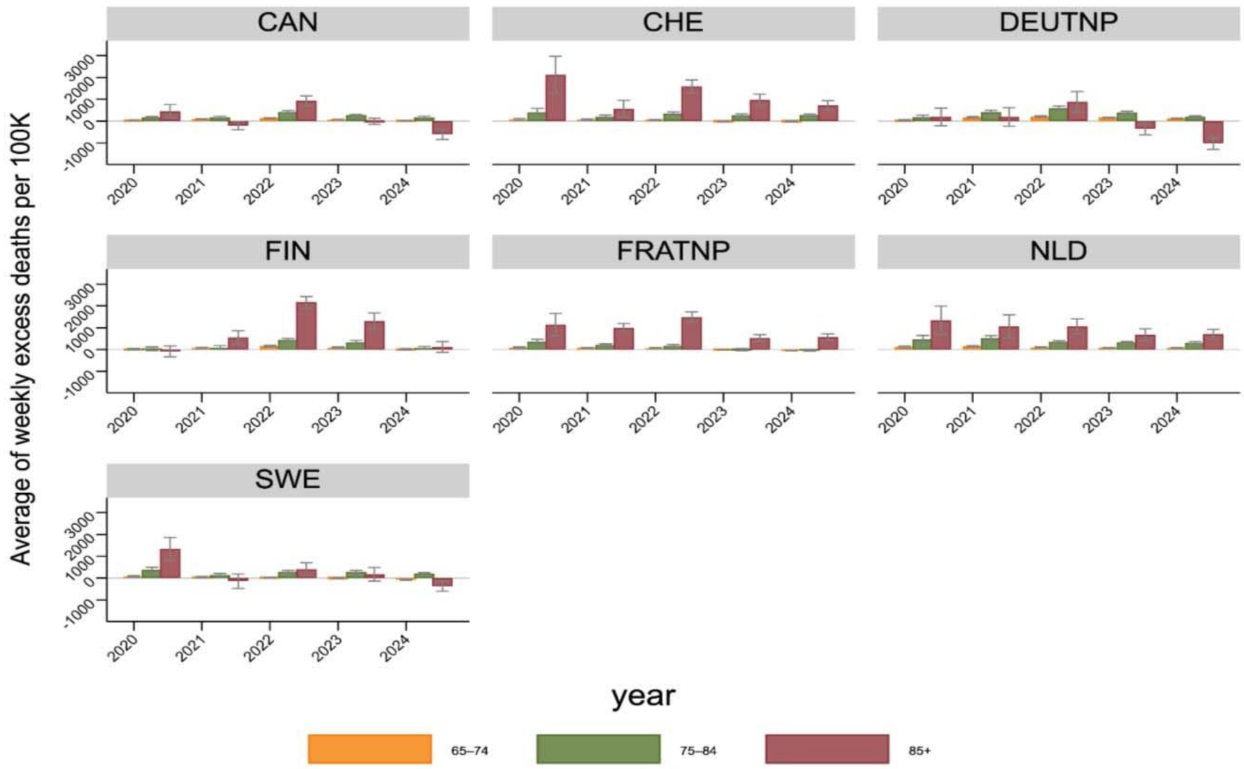

Note: CAN, Canada; CHE, Switzerland; DEUTNP, Germany; FIN, Finland; FRATNP, France; NLD, Netherlands; SWE, Sweden

© 2026 Chen X et al. *JAMA Network Open*.

eFigure 3. Excess Death Rate by Country, Year, and by Age Group for Quintile 3

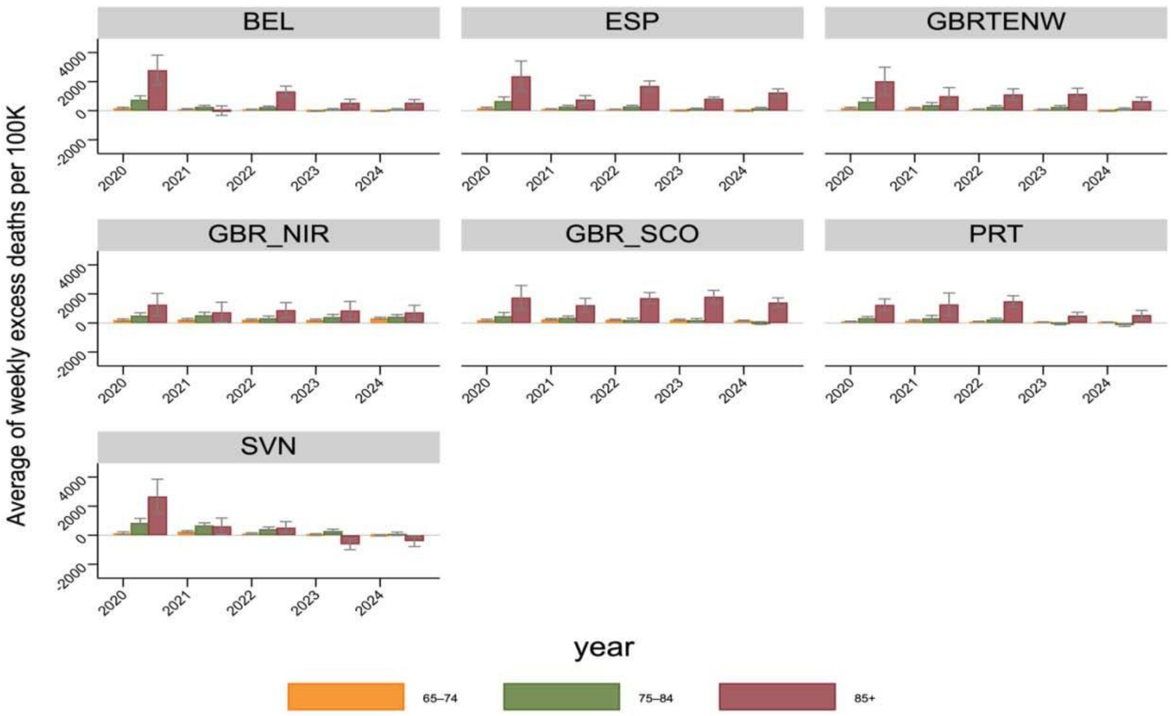

Note: BEL, Belgium; ESP, Spain; GBRTENW, England and Wales; GBR_NIR, Northern Ireland; GBR_SCO, Scotland; PRT, Portugal; SVN, Slovenia

eFigure 4. Excess Death Rate by Country, Year, and by Age Group for Quintile 4

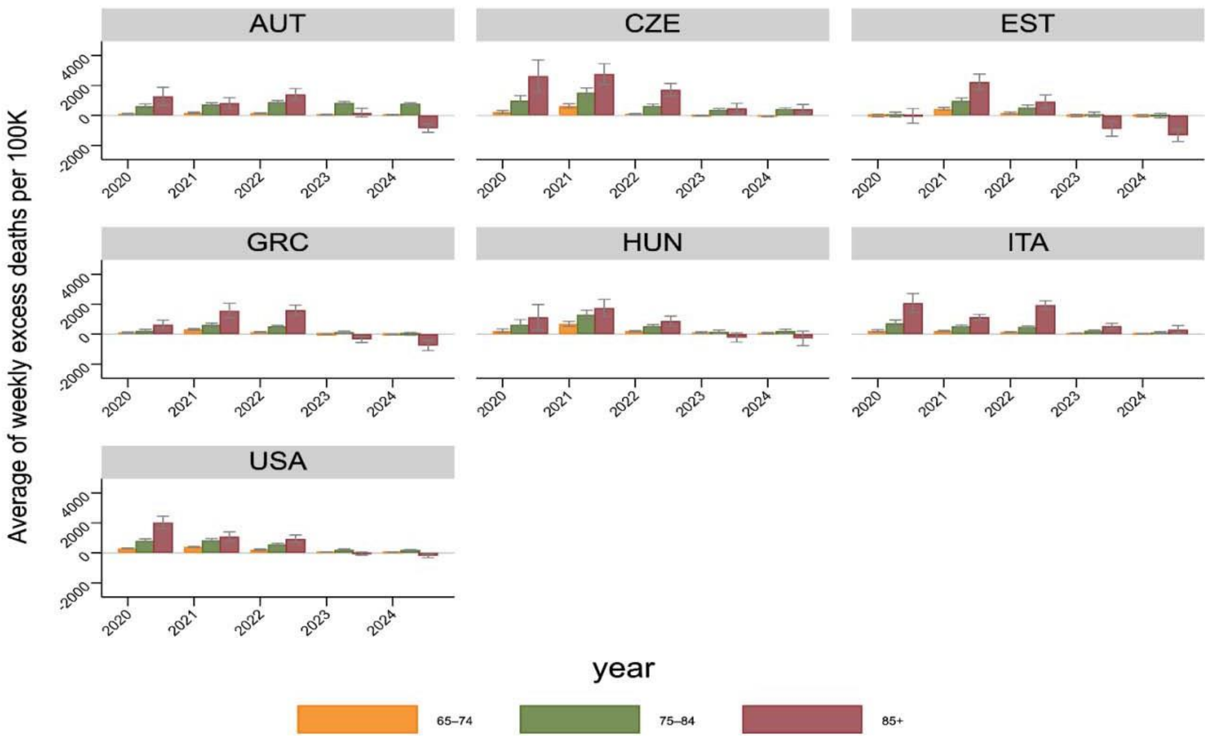

Note: AUT, Austria; CZE, Czechia; EST, Estonia; GRC, Greece; HUN, Hungary; ITA, Italy; USA, United States of America

© 2026 Chen X et al. *JAMA Network Open*.

eFigure 5. Excess Death Rate by Country, Year, and by Age Group for Quintile 5

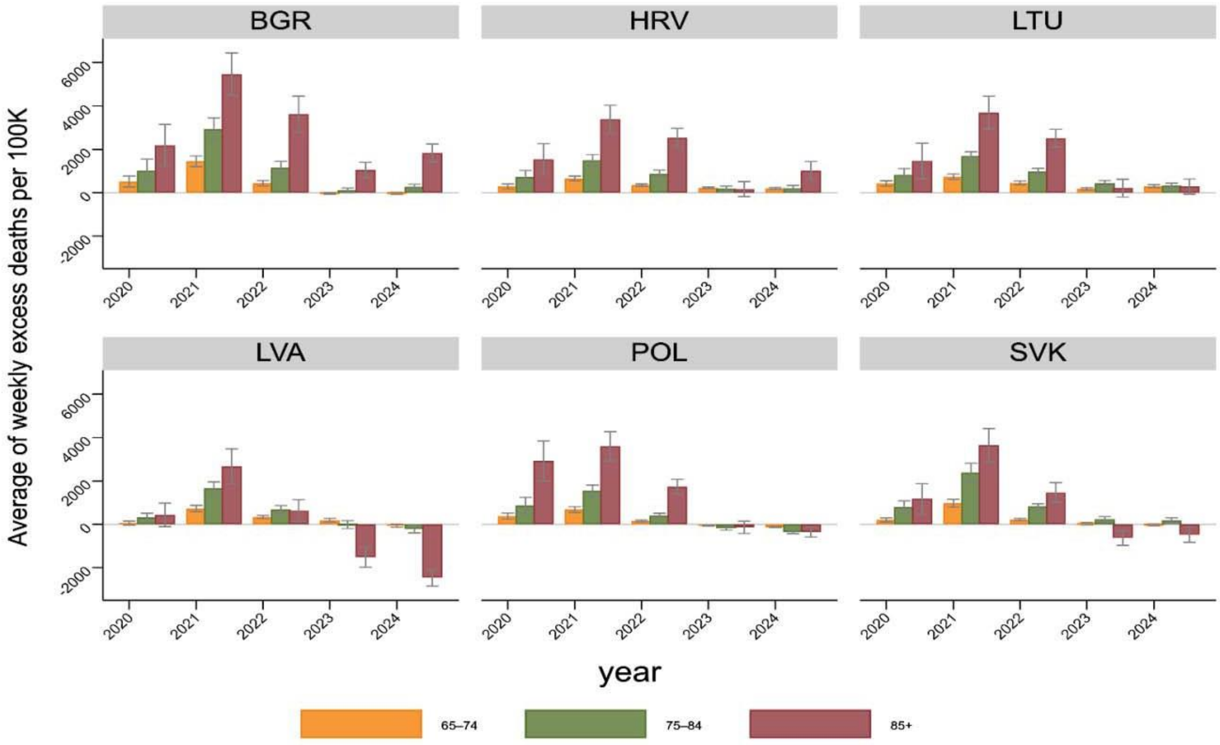

Note: BGR, Bulgaria; HRV, Croatia; LTU, Lithuania; LVA, Latvia; POL, Poland; SVK, Slovakia

© 2026 Chen X et al. *JAMA Network Open*.

eTable 1. Annual Excess Death Rate per 100,000 Population and Estimated Harvesting Effect, by Country and Age Category, 2020-2024, Relative to Expected Deaths Based on 2015-2019 Mortality[d]

Quintiles	Country	Age group	2020	2021	2022	2023	2024	Harvesting effect ratio (%)[e,f]
1	Australia	0-14	-1 (-2, -1)[a]	2 (1, 2)[a]	0 (-0, 1)	-0 (-1, 1)	-0 (-1, 0)	49 (-1088, 1186)
1	Australia	15-64	-2 (-4, 0)	0 (-2, 2)	14 (12, 16)[a]	8 (6, 11)[a]	10 (8, 12)[a]	.
1	Australia	65-74	-24 (-40, -8)[a]	22 (9, 35)[a]	104 (86, 122)[a]	55 (41, 68)[a]	56 (41, 71)[a]	.
1	Australia	75-84	-58 (-101, -16)[a]	111 (74, 148)[a]	442 (390, 494)[a]	278 (234, 321)[a]	230 (192, 268)[a]	.
1	Australia	85over	-456 (-627, -285)[a]	320 (173, 466)[a]	1761 (1551, 1971)[a]	531 (339, 724)[a]	589 (421, 757)[a]	.
1	Denmark	0-14	-0 (-4, 4)	2 (-2, 5)	-0 (-4, 3)	0 (-3, 4)	7 (3, 11)[a]	.
1	Denmark	15-64	-2 (-6, 3)	4 (-1, 10)	6 (1, 11)[b]	5 (0, 9)[b]	-3 (-8, 1)	24 (-66, 115)
1	Denmark	65-74	-36 (-68, -3)[b]	12 (-28, 52)	45 (13, 78)[a]	-47 (-82, -11)[b]	-132 (-167, -96)[a]	802 (-18545, 20149)
1	Denmark	75-84	-80 (-162, 1)[c]	64 (-34, 161)	265 (180, 350)[a]	191 (121, 261)[a]	99 (26, 171)[a]	.
1	Denmark	85over	-470 (-756, -185)[a]	127 (-337, 591)	251 (-8, 509)[c]	-661 (-977, -344)[a]	-1527 (-1796, -1258)[a]	.
1	Iceland	0-14	18 (5, 32)[a]	23 (9, 36)[a]	12 (2, 22)[b]	19 (5, 33)[a]	17 (5, 30)[a]	.
1	Iceland	15-64	-3 (-20, 14)	-9 (-24, 7)	-4 (-20, 12)	5 (-12, 21)	-28 (-42, -14)[a]	.
1	Iceland	65-74	87 (-29, 203)	163 (43, 283)[a]	354 (222, 486)[a]	236 (126, 345)[a]	387 (276, 498)[a]	.
1	Iceland	75-84	-79 (-392, 234)	-302 (-612, 7)[c]	579 (236, 921)[a]	3 (-295, 302)	96 (-198, 391)	.
1	Iceland	85over	-122 (-1148, 904)	558 (-334, 1451)	2246 (1153, 3338)[a]	1744 (762, 2726)[a]	2081 (983, 3179)[a]	.
1	Israel	0-14	-5 (-7, -4)[a]	0 (-2, 3)	1 (-1, 3)	6 (1, 11)[b]	2 (0, 5)[b]	.
1	Israel	15-64	3 (-0, 7)[c]	11 (8, 15)[a]	7 (4, 10)[a]	20 (-3, 43)[c]	13 (9, 17)[a]	.
1	Israel	65-74	100 (63, 136)[a]	149 (110, 188)[a]	111 (78, 144)[a]	33 (5, 62)[b]	78 (52, 104)[a]	.
1	Israel	75-84	255 (148, 361)[a]	276 (192, 360)[a]	188 (91, 285)[a]	-192 (-245, -139)[a]	-111 (-164, -59)[a]	42 (27, 58)[a]
1	Israel	85over	766 (428, 1104)[a]	1004 (684, 1323)[a]	1280 (870, 1690)[a]	-241 (-448, -33)[b]	276 (67, 485)[a]	.
1	Luxembourg	0-14	-4 (-15, 7)	-10 (-21, 2)[c]	-10 (-21, 1)[c]	-7 (-19, 4)	38 (20, 56)[a]	.
1	Luxembourg	15-64	-10 (-23, 3)	-11 (-23, 1)[c]	-17 (-28, -6)[a]	-31 (-43, -20)[a]	-37 (-48, -26)[a]	.
1	Luxembourg	65-74	61 (-53, 174)	81 (-32, 195)	3 (-93, 99)	-48 (-140, 45)	-18 (-119, 84)	45 (-851, 941)
1	Luxembourg	75-84	224 (-73, 521)	52 (-157, 260)	-92 (-305, 121)	-114 (-361, 133)	-191 (-402, 20)[c]	166 (-2392, 2723)
1	Luxembourg	85over	894 (90, 1697)[b]	57 (-585, 699)	-245 (-969, 479)	-666 (-1472, 141)	-1188 (-1825, -551)[a]	263 (-1312, 1837)
1	Norway	0-14	-3 (-6, -0)[b]	-2 (-5, 1)	-1 (-4, 2)	2 (-2, 5)	1 (-2, 4)	.
1	Norway	15-64	-0 (-4, 4)	1 (-3, 5)	11 (6, 17)[a]	11 (7, 14)[a]	9 (5, 13)[a]	.
1	Norway	65-74	-1 (-32, 30)	3 (-37, 43)	71 (38, 104)[a]	11 (-24, 46)	15 (-20, 50)	.
1	Norway	75-84	-93 (-166, -20)[b]	62 (-47, 170)	349 (269, 429)[a]	207 (115, 299)[a]	266 (200, 333)[a]	.
1	Norway	85over	-160 (-464, 144)	440 (22, 859)[b]	1893 (1576, 2210)[a]	595 (309, 881)[a]	445 (152, 738)[a]	.
1	New Zealand - Non-Provincial	0-14	-5 (-6, -4)[a]	-0 (-1, 1)	-4 (-5, -3)[a]	-5 (-6, -4)[a]	-7 (-8, -6)[a]	.
1	New Zealand - Non-Provincial	15-64	-13 (-19, -8)[a]	-9 (-14, -5)[a]	3 (-2, 8)	0 (-5, 6)	-7 (-11, -2)[a]	.
1	New Zealand - Non-Provincial	65-74	-52 (-80, -25)[a]	-7 (-37, 23)	65 (37, 93)[a]	26 (2, 49)[b]	-38 (-63, -13)[a]	121 (-18, 261)[c]
1	New Zealand - Non-Provincial	75-84	-173 (-240, -107)[a]	-23 (-82, 37)	332 (265, 399)[a]	181 (121, 241)[a]	53 (-8, 115)[c]	.
1	New Zealand - Non-Provincial	85over	-1294 (-1637, -950)[a]	-465 (-693, -237)[a]	845 (532, 1157)[a]	-181 (-489, 126)	-834 (-1048, -619)[a]	.
2	Canada	0-14	-0 (-1, 1)	-1 (-1, 0)[c]	4 (3, 5)[a]	4 (3, 5)[a]	2 (1, 3)[a]	.
2	Canada	15-64	17 (14, 20)[a]	27 (23, 30)[a]	24 (21, 27)[a]	15 (12, 17)[a]	-3 (-7, 1)	4 (-1, 8)
2	Canada	65-74	37 (20, 53)[a]	88 (73, 102)[a]	136 (119, 154)[a]	69 (55, 82)[a]	15 (-6, 35)	.
2	Canada	75-84	161 (116, 206)[a]	182 (140, 224)[a]	424 (373, 475)[a]	266 (232, 300)[a]	174 (123, 225)[a]	.
2	Canada	85over	449 (146, 751)[a]	-210 (-397, -23)[b]	929 (700, 1157)[a]	-15 (-158, 128)	-606 (-845, -366)[a]	53 (22, 84)[a]
2	Switzerland	0-14	4 (1, 7)[b]	2 (-1, 5)	5 (2, 8)[a]	2 (-1, 5)	2 (-1, 5)	.
2	Switzerland	15-64	6 (2, 9)[a]	11 (8, 15)[a]	10 (7, 14)[a]	4 (1, 7)[b]	6 (3, 9)[a]	.
2	Switzerland	65-74	73 (26, 120)[a]	59 (27, 90)[a]	45 (18, 71)[a]	-10 (-30, 10)	-8 (-34, 18)	10 (-9, 30)
2	Switzerland	75-84	396 (217, 576)[a]	188 (104, 273)[a]	353 (282, 424)[a]	261 (191, 331)[a]	274 (222, 326)[a]	.

© 2026 Chen X et al. *JAMA Network Open*.

2	Switzerland	85over	2117 (1264, 2970)[a]	564 (174, 954)[a]	1585 (1284, 1885)[a]	961 (686, 1237)[a]	716 (502, 930)[a]	.
	Germany -		-2 (-3, -2)[a]	-1 (-2, -0)[a]	1 (-0, 2)[c]	1 (-0, 1)[c]	2 (1, 3)[a]	.
2	Total National Population	0-14	5 (3, 7)[a]	22 (18, 27)[a]	23 (19, 27)[a]	18 (15, 22)[a]	13 (11, 16)[a]	.
	Germany -		34 (7, 61)[b]	167 (132, 202)[a]	199 (164, 235)[a]	160 (137, 184)[a]	114 (97, 132)[a]	.
2	Total National Population	15-64	184 (102, 267)[a]	407 (315, 499)[a]	590 (497, 683)[a]	393 (329, 458)[a]	193 (148, 239)[a]	.
	Germany -		190 (-211, 591)	188 (-233, 609)	882 (417, 1347)[a]	-356 (-627, -86)[a]	-1018 (-1300, -736)[a]	109 (-41, 259)
2	Total National Population	65-74	-3 (-6, -1)[b]	-1 (-3, 2)	0 (-3, 4)	-1 (-3, 2)	-0 (-3, 2)	.
	Germany -		7 (2, 12)[a]	7 (1, 13)[b]	13 (7, 19)[a]	11 (5, 16)[a]	1 (-5, 7)	.
2	Total National Population	75-84	15 (-17, 47)	69 (32, 106)[a]	162 (129, 194)[a]	81 (47, 115)[a]	13 (-16, 42)	.
	Germany -		41 (-42, 125)	92 (6, 178)[b]	447 (376, 517)[a]	318 (216, 420)[a]	74 (5, 143)[b]	.
2	Total National Population	85over	-79 (-329, 170)	555 (242, 867)[a]	2172 (1916, 2428)[a]	1308 (940, 1677)[a]	114 (-131, 359)	.
2	Finland	0-14	-2 (-3, -1)[a]	-1 (-3, -0)[b]	1 (0, 3)[a]	-0 (-2, 1)	1 (-0, 2)	.
2	Finland	15-64	6 (2, 9)[a]	1 (-2, 5)	3 (-0, 6)[c]	-4 (-6, -1)[a]	-4 (-7, -2)[a]	81 (-431, 593)
2	Finland	65-74	87 (54, 120)[a]	78 (59, 97)[a]	62 (42, 82)[a]	-13 (-25, -1)[b]	-38 (-50, -27)[a]	23 (14, 31)[a]
2	Finland	75-84	352 (227, 477)[a]	211 (161, 261)[a]	174 (115, 232)[a]	-3 (-49, 43)	-32 (-71, 8)	5 (-2, 12)
2	Finland	85over	1142 (635, 1649)[a]	998 (805, 1191)[a]	1492 (1259, 1724)[a]	526 (367, 686)[a]	574 (425, 723)[a]	.
	France -		1 (-1, 3)	-1 (-3, 1)	-1 (-3, 1)	-0 (-3, 2)	-1 (-3, 1)	.
2	Total National Population	0-14	7 (3, 10)[a]	19 (16, 23)[a]	15 (12, 18)[a]	11 (8, 14)[a]	14 (12, 17)[a]	.
	France -		109 (66, 152)[a]	148 (116, 179)[a]	94 (69, 120)[a]	82 (62, 103)[a]	72 (54, 90)[a]	.
2	Total National Population	15-64	473 (303, 643)[a]	523 (407, 638)[a]	348 (282, 414)[a]	320 (268, 371)[a]	309 (263, 355)[a]	.
	France -		1333 (675, 1991)[a]	1064 (532, 1596)[a]	1063 (722, 1403)[a]	679 (410, 948)[a]	705 (492, 918)[a]	.
2	Total National Population	65-74	3 (1, 5)[a]	1 (-1, 3)	4 (1, 6)[a]	4 (2, 6)[a]	5 (3, 7)[a]	.
	France -		8 (4, 13)[a]	11 (8, 13)[a]	9 (6, 12)[a]	12 (8, 15)[a]	7 (5, 10)[a]	.
2	Total National Population	75-84	67 (28, 105)[a]	39 (10, 68)[a]	18 (-9, 45)	-2 (-28, 24)	-74 (-96, -53)[a]	62 (16, 108)[a]
	France -		376 (253, 499)[a]	149 (82, 216)[a]	281 (215, 347)[a]	287 (212, 362)[a]	206 (157, 255)[a]	.
2	Total National Population	85over	1341 (817, 1866)[a]	-140 (-474, 194)	402 (104, 700)[a]	171 (-138, 481)	-369 (-604, -134)[a]	21 (1, 41)[b]
2	Netherlands	0-14	-4 (-6, -2)[a]	-4 (-7, -2)[a]	-5 (-7, -3)[a]	-11 (-13, -9)[a]	-11 (-13, -9)[a]	.
2	Netherlands	15-64	15 (9, 21)[a]	15 (10, 20)[a]	14 (10, 18)[a]	10 (6, 13)[a]	11 (7, 15)[a]	.
2	Netherlands	65-74	171 (100, 241)[a]	104 (64, 144)[a]	71 (42, 100)[a]	-22 (-48, 4)[c]	-24 (-48, 0)[c]	13 (2, 25)[b]
2	Netherlands	75-84	751 (480, 1022)[a]	267 (174, 359)[a]	247 (180, 315)[a]	88 (27, 149)[a]	85 (22, 148)[a]	.
2	Netherlands	85over	2790 (1761, 3818)[a]	4 (-323, 330)	1326 (966, 1687)[a]	548 (309, 787)[a]	550 (331, 769)[a]	.
2	Sweden	0-14	-2 (-3, -1)[a]	-1 (-2, 1)	2 (1, 3)[a]	1 (0, 2)[b]	4 (2, 5)[a]	.
2	Sweden	15-64	14 (9, 19)[a]	11 (9, 14)[a]	8 (5, 10)[a]	-3 (-5, -1)[a]	-6 (-8, -4)[a]	28 (17, 39)[a]
2	Sweden	65-74	173 (97, 248)[a]	115 (93, 136)[a]	90 (71, 108)[a]	24 (13, 34)[a]	2 (-16, 21)	.
2	Sweden	75-84	665 (383, 947)[a]	285 (204, 366)[a]	294 (221, 367)[a]	135 (93, 176)[a]	168 (110, 226)[a]	.
2	Sweden	85over	2374 (1333, 3416)[a]	767 (492, 1042)[a]	1703 (1354, 2051)[a]	823 (709, 937)[a]	1252 (1000, 1504)[a]	.
3	Belgium	0-14	-2 (-3, -1)[a]	0 (-1, 1)	2 (1, 3)[a]	4 (3, 5)[a]	5 (3, 6)[a]	.
3	Belgium	15-64	23 (15, 31)[a]	32 (25, 39)[a]	15 (11, 20)[a]	18 (13, 22)[a]	9 (5, 13)[a]	.
3	Belgium	65-74	170 (98, 242)[a]	163 (99, 226)[a]	87 (57, 118)[a]	69 (38, 101)[a]	1 (-27, 29)	.
3	Belgium	75-84	625 (374, 876)[a]	381 (205, 557)[a]	260 (170, 350)[a]	264 (173, 356)[a]	122 (52, 192)[a]	.
3	Belgium	85over	2030 (1072, 2989)[a]	1002 (416, 1589)[a]	1126 (745, 1508)[a]	1161 (775, 1547)[a]	666 (402, 930)[a]	.
3	Spain	0-14	-5 (-10, 1)	4 (-1, 10)	2 (-4, 8)	-2 (-8, 3)	3 (-3, 9)	.
3	Spain	15-64	19 (8, 30)[a]	28 (17, 39)[a]	8 (-4, 19)	4 (-9, 17)	29 (15, 44)[a]	.
3	Spain	65-74	201 (129, 273)[a]	231 (152, 309)[a]	212 (146, 277)[a]	201 (132, 269)[a]	317 (242, 391)[a]	.
3	Spain	75-84	506 (310, 702)[a]	524 (317, 731)[a]	322 (166, 478)[a]	413 (236, 591)[a]	432 (286, 578)[a]	.

© 2026 Chen X et al. *JAMA Network Open*.

3	Spain	85over	1264 (498, 2031)[a]	739 (50, 1428)[b]	888 (373, 1403)[a]	860 (242, 1478)[a]	728 (245, 1212)[a]	.
3	England and Wales	0-14	-2 (-5, 2)	3 (-1, 7)	1 (-2, 4)	6 (2, 9)[a]	3 (-1, 6)	.
3	England and Wales	15-64	19 (9, 29)[a]	28 (19, 37)[a]	-1 (-10, 7)	-1 (-9, 7)	-13 (-20, -6)[a]	31 (6, 57)[b]
3	England and Wales	65-74	184 (113, 256)[a]	250 (194, 306)[a]	216 (168, 264)[a]	211 (159, 263)[a]	152 (109, 195)[a]	.
3	England and Wales	75-84	472 (222, 721)[a]	357 (235, 479)[a]	202 (91, 313)[a]	190 (76, 304)[a]	-6 (-101, 90)	0 (-5, 6)
3	England and Wales	85over	1750 (923, 2576)[a]	1230 (765, 1696)[a]	1712 (1335, 2090)[a]	1813 (1380, 2246)[a]	1412 (1097, 1727)[a]	.
3	Northern Ireland	0-14	-5 (-8, -2)[a]	-5 (-8, -2)[a]	-3 (-5, 0)[c]	-3 (-6, 0)[c]	-4 (-7, -2)[a]	.
3	Northern Ireland	15-64	13 (7, 18)[a]	15 (8, 22)[a]	9 (5, 13)[a]	-1 (-6, 4)	-8 (-13, -3)[a]	25 (7, 43)[a]
3	Northern Ireland	65-74	94 (62, 126)[a]	147 (83, 211)[a]	96 (68, 123)[a]	59 (39, 79)[a]	48 (22, 74)[a]	.
3	Northern Ireland	75-84	331 (219, 443)[a]	321 (122, 519)[a]	238 (154, 322)[a]	-51 (-124, 21)	-148 (-238, -57)[a]	22 (7, 38)[a]
3	Northern Ireland	85over	1251 (845, 1657)[a]	1280 (501, 2059)[a]	1493 (1114, 1871)[a]	509 (286, 732)[a]	548 (226, 869)[a]	.
3	Scotland	0-14	-2 (-7, 3)	-2 (-6, 2)	2 (-3, 7)	-3 (-8, 2)	-0 (-5, 5)	.
3	Scotland	15-64	4 (-6, 14)	28 (16, 39)[a]	18 (10, 25)[a]	10 (1, 20)[b]	5 (-4, 13)	.
3	Scotland	65-74	135 (39, 231)[a]	245 (173, 317)[a]	115 (54, 175)[a]	46 (-13, 106)	-4 (-51, 43)	1 (-6, 7)
3	Scotland	75-84	848 (541, 1156)[a]	676 (498, 854)[a]	421 (277, 564)[a]	289 (158, 419)[a]	97 (-19, 214)	.
3	Scotland	85over	2672 (1485, 3859)[a]	616 (46, 1185)[b]	527 (120, 934)[b]	-629 (-995, -263)[a]	-407 (-783, -32)[b]	27 (9, 46)[a]
3	Portugal	0-14	1 (-2, 4)	-2 (-5, 1)	-3 (-5, -0)[b]	-1 (-4, 2)	0 (-3, 3)	.
3	Portugal	15-64	10 (5, 15)[a]	23 (18, 29)[a]	21 (16, 27)[a]	13 (9, 18)[a]	14 (10, 19)[a]	.
3	Portugal	65-74	99 (53, 145)[a]	171 (127, 215)[a]	144 (108, 180)[a]	57 (24, 91)[a]	40 (9, 71)[b]	.
3	Portugal	75-84	609 (456, 763)[a]	736 (626, 846)[a]	890 (794, 987)[a]	838 (752, 924)[a]	790 (726, 853)[a]	.
3	Portugal	85over	1276 (671, 1881)[a]	816 (453, 1179)[a]	1412 (1026, 1798)[a]	174 (-113, 461)	-850 (-1134, -566)[a]	23 (13, 33)[a]
3	Slovenia	0-14	-4 (-6, -2)[a]	-3 (-6, -1)[a]	-4 (-6, -1)[a]	-6 (-8, -3)[a]	-5 (-7, -3)[a]	.
3	Slovenia	15-64	17 (8, 25)[a]	65 (51, 79)[a]	11 (6, 15)[a]	-0 (-5, 4)	-0 (-5, 5)	1 (-4, 5)
3	Slovenia	65-74	236 (139, 334)[a]	632 (494, 770)[a]	95 (56, 135)[a]	-25 (-57, 7)	-57 (-85, -29)[a]	8 (4, 13)[a]
3	Slovenia	75-84	986 (651, 1322)[a]	1533 (1225, 1841)[a]	647 (538, 756)[a]	364 (271, 458)[a]	430 (357, 503)[a]	.
3	Slovenia	85over	2624 (1544, 3704)[a]	2771 (2089, 3454)[a]	1719 (1302, 2136)[a]	455 (102, 809)[b]	423 (115, 732)[a]	.
4	Austria	0-14	-2 (-8, 3)	6 (-1, 13)	7 (-0, 14)[c]	8 (2, 15)[b]	4 (-2, 9)	.
4	Austria	15-64	25 (13, 38)[a]	70 (55, 84)[a]	58 (44, 73)[a]	53 (39, 67)[a]	65 (52, 79)[a]	.
4	Austria	65-74	-4 (-88, 81)	442 (347, 537)[a]	159 (83, 236)[a]	-11 (-88, 66)	-31 (-112, 49)	7 (-9, 23)
4	Austria	75-84	59 (-98, 216)	972 (771, 1173)[a]	541 (385, 698)[a]	72 (-86, 229)	-15 (-170, 139)	1 (-6, 7)
4	Austria	85over	-37 (-526, 451)	2237 (1712, 2762)[a]	916 (461, 1370)[a]	-897 (-1396, -397)[a]	-1326 (-1737, -915)[a]	71 (41, 101)[a]
4	Czechia	0-14	-2 (-5, 0)[c]	1 (-1, 4)	-2 (-4, 1)	-0 (-3, 2)	4 (1, 7)[a]	.
4	Czechia	15-64	7 (1, 13)[b]	47 (38, 56)[a]	19 (14, 25)[a]	3 (-2, 9)	-7 (-12, -2)[a]	9 (2, 16)[a]
4	Czechia	65-74	102 (57, 146)[a]	311 (256, 367)[a]	130 (93, 167)[a]	0 (-26, 26)	-11 (-39, 18)	2 (-3, 7)
4	Czechia	75-84	227 (130, 324)[a]	611 (480, 741)[a]	513 (444, 582)[a]	138 (75, 201)[a]	14 (-75, 102)	.
4	Czechia	85over	641 (357, 924)[a]	1572 (1087, 2058)[a]	1609 (1275, 1944)[a]	-358 (-560, -157)[a]	-758 (-1101, -416)[a]	29 (18, 41)[a]
4	Estonia	0-14	-0 (-3, 2)	1 (-3, 4)	5 (2, 8)[a]	3 (-1, 6)	7 (4, 11)[a]	.
4	Estonia	15-64	16 (4, 29)[b]	103 (77, 130)[a]	10 (4, 15)[a]	-16 (-23, -10)[a]	-18 (-25, -11)[a]	26 (17, 36)[a]
4	Estonia	65-74	224 (104, 345)[a]	694 (531, 857)[a]	205 (167, 243)[a]	111 (69, 154)[a]	63 (18, 109)[a]	.
4	Estonia	75-84	633 (298, 967)[a]	1317 (1040, 1593)[a]	540 (444, 635)[a]	171 (66, 277)[a]	204 (77, 330)[a]	.
4	Estonia	85over	1122 (267, 1977)[b]	1746 (1170, 2321)[a]	879 (550, 1208)[a]	-226 (-536, 83)	-278 (-757, 201)	13 (-4, 31)
4	Greece	0-14	-2 (-3, -1)[a]	-1 (-2, -0)[b]	-2 (-3, -1)[a]	1 (-0, 1)	1 (-0, 2)	.
4	Greece	15-64	18 (13, 23)[a]	24 (20, 28)[a]	15 (12, 18)[a]	9 (7, 11)[a]	6 (4, 9)[a]	.
4	Greece	65-74	228 (159, 297)[a]	216 (181, 250)[a]	139 (121, 157)[a]	66 (52, 81)[a]	31 (16, 45)[a]	.
4	Greece	75-84	731 (516, 946)[a]	539 (473, 606)[a]	497 (442, 551)[a]	231 (190, 272)[a]	119 (71, 166)[a]	.
4	Greece	85over	2080 (1443, 2716)[a]	1136 (946, 1326)[a]	1950 (1681, 2219)[a]	545 (371, 719)[a]	298 (30, 566)[b]	.
4	Hungary	0-14	-1 (-2, -1)[a]	2 (1, 2)[a]	5 (5, 6)[a]	6 (5, 6)[a]	6 (6, 7)[a]	.
4	Hungary	15-64	56 (47, 64)[a]	100 (88, 111)[a]	44 (37, 51)[a]	10 (8, 12)[a]	-14 (-16, -12)[a]	7 (6, 8)[a]
4	Hungary	65-74	287 (239, 335)[a]	395 (348, 443)[a]	212 (171, 253)[a]	66 (57, 76)[a]	36 (24, 47)[a]	.
4	Hungary	75-84	807 (681, 933)[a]	847 (734, 960)[a]	564 (480, 648)[a]	224 (195, 252)[a]	191 (164, 219)[a]	.
4	Hungary	85over	2040 (1626, 2454)[a]	1085 (774, 1396)[a]	944 (696, 1192)[a]	-58 (-160, 44)	-216 (-325, -108)[a]	7 (3, 10)[a]
4	Italy	0-14	-7 (-11, -3)[a]	3 (-2, 8)	-3 (-7, 1)	2 (-3, 6)	-3 (-8, 1)	.
4	Italy	15-64	61 (25, 96)[a]	190 (158, 221)[a]	29 (15, 42)[a]	-25 (-32, -17)[a]	-21 (-29, -12)[a]	16 (11, 21)[a]

© 2026 Chen X et al. *JAMA Network Open*.

4	Italy	65-74	518 (263, 774)[a]	1446 (1201, 1691)[a]	435 (309, 562)[a]	-34 (-70, 3)[c]	-30 (-82, 23)	3 (-0, 6)[c]
4	Italy	75-84	1009 (470, 1549)[a]	2929 (2411, 3447)[a]	1149 (857, 1441)[a]	109 (7, 212)[b]	275 (161, 388)[a]	.
4	Italy	85over	2186 (1227, 3146)[a]	5465 (4489, 6442)[a]	3627 (2805, 4450)[a]	1052 (705, 1399)[a]	1829 (1413, 2244)[a]	.
4	United States of America (USA)	0-14	0 (-5, 5)	1 (-3, 6)	2 (-2, 7)	3 (-2, 8)	-3 (-8, 2)	39 (-936, 1014)
4	United States of America (USA)	15-64	11 (-1, 22)[c]	54 (40, 68)[a]	8 (-1, 18)[c]	-1 (-8, 5)	-15 (-22, -8)[a]	22 (9, 36)[a]
4	United States of America (USA)	65-74	283 (166, 400)[a]	651 (538, 764)[a]	352 (298, 406)[a]	221 (180, 263)[a]	198 (152, 243)[a]	.
4	United States of America (USA)	75-84	728 (425, 1030)[a]	1500 (1240, 1760)[a]	864 (689, 1039)[a]	181 (55, 308)[a]	200 (66, 335)[a]	.
4	United States of America (USA)	85over	1540 (818, 2263)[a]	3386 (2738, 4034)[a]	2539 (2116, 2961)[a]	163 (-183, 510)	1019 (600, 1437)[a]	.
5	Bulgaria	0-14	-0 (-6, 5)	5 (-0, 11)[c]	1 (-3, 6)	6 (1, 11)[b]	2 (-2, 7)	.
5	Bulgaria	15-64	81 (61, 100)[a]	140 (121, 159)[a]	78 (64, 92)[a]	64 (48, 80)[a]	78 (64, 93)[a]	.
5	Bulgaria	65-74	423 (302, 544)[a]	743 (626, 860)[a]	450 (365, 536)[a]	180 (122, 237)[a]	295 (227, 363)[a]	.
5	Bulgaria	75-84	818 (525, 1110)[a]	1688 (1491, 1884)[a]	973 (826, 1120)[a]	433 (304, 563)[a]	334 (225, 443)[a]	.
5	Bulgaria	85over	1468 (659, 2277)[a]	3685 (2928, 4443)[a]	2510 (2100, 2920)[a]	211 (-196, 619)	286 (-64, 637)	.
5	Croatia	0-14	4 (-2, 10)	-2 (-9, 5)	3 (-3, 8)	0 (-5, 6)	-3 (-8, 2)	60 (-2838, 2958)
5	Croatia	15-64	-6 (-21, 9)	132 (106, 158)[a]	71 (52, 89)[a]	28 (12, 43)[a]	-21 (-37, -5)[a]	9 (2, 17)[b]
5	Croatia	65-74	58 (-35, 151)	726 (576, 876)[a]	342 (268, 415)[a]	188 (101, 275)[a]	-58 (-129, 13)	4 (-1, 10)
5	Croatia	75-84	321 (128, 514)[a]	1661 (1370, 1952)[a]	684 (509, 858)[a]	-15 (-196, 166)	-219 (-392, -47)[b]	9 (0, 17)[b]
5	Croatia	85over	436 (-102, 974)	2675 (1868, 3481)[a]	634 (126, 1141)[b]	-1517 (-1975, -1059)[a]	-2460 (-2842, -2079)[a]	106 (68, 144)[a]
5	Lithuania	0-14	-4 (-6, -3)[a]	-1 (-3, 0)[c]	-1 (-2, 1)	-3 (-5, -1)[a]	-6 (-7, -4)[a]	.
5	Lithuania	15-64	20 (4, 36)[b]	71 (55, 86)[a]	9 (2, 16)[a]	-28 (-32, -23)[a]	-41 (-45, -36)[a]	68 (49, 88)[a]
5	Lithuania	65-74	381 (239, 523)[a]	681 (548, 815)[a]	160 (118, 202)[a]	-64 (-88, -41)[a]	-130 (-153, -107)[a]	16 (12, 20)[a]
5	Lithuania	75-84	865 (483, 1247)[a]	1544 (1285, 1804)[a]	411 (314, 508)[a]	-178 (-268, -88)[a]	-350 (-430, -271)[a]	19 (13, 24)[a]
5	Lithuania	85over	2921 (1996, 3846)[a]	3602 (2930, 4275)[a]	1740 (1403, 2077)[a]	-136 (-415, 144)	-354 (-586, -122)[a]	6 (2, 10)[a]
5	Latvia	0-14	0 (-4, 5)	-2 (-7, 2)	1 (-3, 6)	-0 (-5, 4)	-2 (-7, 2)	.
5	Latvia	15-64	11 (2, 20)[b]	115 (92, 137)[a]	24 (17, 32)[a]	-2 (-8, 5)	-6 (-12, -0)[b]	5 (-0, 11)[c]
5	Latvia	65-74	206 (112, 300)[a]	969 (787, 1151)[a]	221 (168, 274)[a]	31 (-21, 83)	-20 (-62, 22)	1 (-1, 4)
5	Latvia	75-84	803 (527, 1079)[a]	2380 (1948, 2811)[a]	820 (688, 952)[a]	238 (122, 353)[a]	176 (47, 305)[a]	.
5	Latvia	85over	1181 (492, 1871)[a]	3646 (2873, 4419)[a]	1467 (1007, 1927)[a]	-623 (-970, -276)[a]	-478 (-832, -125)[a]	18 (9, 26)[a]
5	Poland	0-14	-1 (-2, -1)[a]	2 (1, 2)[a]	0 (-0, 1)	-0 (-1, 1)	-0 (-1, 0)	49 (-1088, 1186)
5	Poland	15-64	-2 (-4, 0)	0 (-2, 2)	14 (12, 16)[a]	8 (6, 11)[a]	10 (8, 12)[a]	.
5	Poland	65-74	-24 (-40, -8)[a]	22 (9, 35)[a]	104 (86, 122)[a]	55 (41, 68)[a]	56 (41, 71)[a]	.
5	Poland	75-84	-58 (-101, -16)[a]	111 (74, 148)[a]	442 (390, 494)[a]	278 (234, 321)[a]	230 (192, 268)[a]	.
5	Poland	85over	-456 (-627, -285)[a]	320 (173, 466)[a]	1761 (1551, 1971)[a]	531 (339, 724)[a]	589 (421, 757)[a]	.
5	Slovakia	0-14	-0 (-4, 4)	2 (-2, 5)	-0 (-4, 3)	0 (-3, 4)	7 (3, 11)[a]	.
5	Slovakia	15-64	-2 (-6, 3)	4 (-1, 10)	6 (1, 11)[b]	5 (0, 9)[b]	-3 (-8, 1)	24 (-66, 115)
5	Slovakia	65-74	-36 (-68, -3)[b]	12 (-28, 52)	45 (13, 78)[a]	-47 (-82, -11)[b]	-132 (-167, -96)[a]	802 (-18545, 20149)
5	Slovakia	75-84	-80 (-162, 1)[c]	64 (-34, 161)	265 (180, 350)[a]	191 (121, 261)[a]	99 (26, 171)[a]	.
5	Slovakia	85over	-470 (-756, -185)[a]	127 (-337, 591)	251 (-8, 509)[c]	-661 (-977, -344)[a]	-1527 (-1796, -1258)[a]	.

Note:

a. p<0.01; b. p<0.05; c. p<0.1; d. Excess deaths per 100k people are shown above;

e. Harvesting effect ratio was defined as the proportion of cumulative excess mortality in 2020-2022 or 2020-2023 that was offset by cumulative negative excess mortality during 2023 and 2024 combined or 2024 alone;

f. A harvesting percentage exceeding 100% indicates that the subsequent mortality deficit entirely offset or surpassed the initial mortality surge, while missing values indicate that the required pattern of all-positive surge years followed by all-negative deficit years was not observed in that group, and therefore no harvesting effect was computed.

© 2026 Chen X et al. *JAMA Network Open*.

eTable 2. Annual Excess Death Rate per 100,000 Population and Estimated Harvesting Effect, by Country, Age Group, and Sex, 2020-2024, Relative to Expected Deaths Based on 2015-2019 Mortality[a,c,d]

Quintile	Country	Age group	Sex	2020	2021	2022	2023	2024	Harvesting effect ratio(%)[b]
1	Australia	0-14	female	-0.17	2.99	1.61	1.57	0.7	
1	Australia	0-14	male	-2.37	0.51	-0.82	-1.71	-1.18	
1	Australia	15-64	female	-0.65	0.72	12.83	8.76	7.87	
1	Australia	15-64	male	-2.59	-0.26	14.97	8.2	12.46	
1	Australia	65-74	female	-30.06	18.59	64.85	31.86	31.71	
1	Australia	65-74	male	-16.7	25.6	146.37	79.13	82.79	
1	Australia	75-84	female	-44.63	89.67	341.89	241.54	208.96	
1	Australia	75-84	male	-73.86	134.75	553.83	317.69	253.76	
1	Australia	85over	female	-466.07	332.56	1685.31	516.35	612.53	
1	Australia	85over	male	-439.46	299.19	1877.83	554.08	554.07	
1	Denmark	0-14	female	0.35	6.11	5.24	5.53	11.17	
1	Denmark	0-14	male	-0.46	-2.43	-5.35	-4.44	3.06	
1	Denmark	15-64	female	3.29	8.37	7.61	4.9	0.77	
1	Denmark	15-64	male	-6.86	0.38	5.31	4.29	-7.3	
1	Denmark	65-74	female	-32.11	12.07	74.48	-34.19	-55.02	163.89
1	Denmark	65-74	male	-39.1	12.48	14.5	-60.23	-213.2	
1	Denmark	75-84	female	-47.43	74.47	248.69	134.97	155.24	
1	Denmark	75-84	male	-119.56	51.44	285.13	256.94	32.5	
1	Denmark	85over	female	-682.07	51.6	177.83	-562.18	-1655.81	
1	Denmark	85over	male	-84.07	262.83	377.88	-829.36	-1311.33	384.58
1	Iceland	0-14	female	6.11	25.54	17.71	15.77	10.88	
1	Iceland	0-14	male	30.01	20	7.23	22.28	23.6	
1	Iceland	15-64	female	11.87	-0.07	6.05	28.81	-4.73	10.14
1	Iceland	15-64	male	-17.12	-16.83	-12.9	-17.33	-49.72	
1	Iceland	65-74	female	130.45	251.46	448.86	232.84	399.23	
1	Iceland	65-74	male	43.16	74.64	258.77	238.75	374.31	
1	Iceland	75-84	female	-363.5	-386.79	335.14	-234.15	0.07	
1	Iceland	75-84	male	237.42	-209.77	841.74	261.25	200.49	
1	Iceland	85over	female	293.55	598.79	2371.6	250.42	1083.61	
1	Iceland	85over	male	-766.66	496.69	2059.64	3905.93	3519.43	
1	Israel	0-14	female	-4.42	0.03	1.8	5.48	1.05	
1	Israel	0-14	male	-6.34	0.68	-0.38	6.22	3.76	
1	Israel	15-64	female	1.37	6.04	4.93	8.74	5.71	
1	Israel	15-64	male	5.58	16.2	8.78	31.27	20.83	
1	Israel	65-74	female	23.33	79.3	35.12	4.83	26.67	
1	Israel	65-74	male	188.19	230.35	198.56	65.91	136.98	
1	Israel	75-84	female	149.85	216.78	87.76	-232.08	-175.58	89.72
1	Israel	75-84	male	390.97	352.67	316.15	-141.28	-29.69	16.13
1	Israel	85over	female	655.06	956.93	1006.92	-535.68	-76.31	23.37
1	Israel	85over	male	947.23	1079.09	1720.84	233.44	839.68	
1	Luxembourg	0-14	female	3.66	-2.07	-16.06	4.88	21.65	
1	Luxembourg	0-14	male	-11.37	-17.09	-4	-19.27	53.18	
1	Luxembourg	15-64	female	-17.81	-10.68	-21.9	-19.15	-27.91	
1	Luxembourg	15-64	male	-2.52	-10.95	-13.14	-42.85	-46.3	

© 2026 Chen X et al. *JAMA Network Open*.

**1**	Luxembourg	65-74	female	95.4	111.76	132.27	79.45	97.32	
**1**	Luxembourg	65-74	male	24.62	49.47	-132.66	-181.2	-137.92	
**1**	Luxembourg	75-84	female	42.09	-145.95	-230.36	-149.76	-335.33	
**1**	Luxembourg	75-84	male	456.84	300.03	80.56	-70.29	-16.04	10.31
**1**	Luxembourg	85over	female	857.34	478.45	580.46	309.19	-559.07	25.12
**1**	Luxembourg	85over	male	966.58	-780.02	-1837.39	-2503.57	-2352.03	
**1**	Norway	0-14	female	-2.34	-1.33	-2.85	0.56	2.57	
**1**	Norway	0-14	male	-4.02	-2.8	1.1	2.47	-0.91	
**1**	Norway	15-64	female	0.55	1.25	9.21	12.38	6.72	
**1**	Norway	15-64	male	-0.74	1.24	13.34	8.79	11.35	
**1**	Norway	65-74	female	-25.58	-19.11	21.88	-44.97	-45.12	
**1**	Norway	65-74	male	24.39	25.14	120.4	67.92	76.21	
**1**	Norway	75-84	female	-105.09	50.31	211.37	122	173.01	
**1**	Norway	75-84	male	-77.94	75.48	506.19	303.06	371.54	
**1**	Norway	85over	female	-256.69	478.64	1639.87	512.14	368.64	
**1**	Norway	85over	male	17.7	370.94	2338.41	737.94	573.72	
**1**	New Zealand - Non-Provincial	0-14	female	-8.27	-1.39	-5.31	-6.21	-7.67	
**1**	New Zealand - Non-Provincial	0-14	male	-1.56	0.89	-2.31	-3.72	-5.94	
**1**	New Zealand - Non-Provincial	15-64	female	-4.86	-2.15	7.91	8.53	5.84	
**1**	New Zealand - Non-Provincial	15-64	male	-21.81	-16.55	-2.3	-7.92	-19.06	
**1**	New Zealand - Non-Provincial	65-74	female	-46.26	-19.8	36.46	7.79	-38.36	
**1**	New Zealand - Non-Provincial	65-74	male	-58.87	6.71	95.85	45	-38.27	43.16
**1**	New Zealand - Non-Provincial	75-84	female	-159.12	97.97	442.66	307.82	252.1	
**1**	New Zealand - Non-Provincial	75-84	male	-190.1	-161.64	205.19	36.19	-172.46	
**1**	New Zealand - Non-Provincial	85over	female	-1284.74	-579.03	840.74	-364.1	-892.13	
**1**	New Zealand - Non-Provincial	85over	male	-1308.42	-287.48	850.79	96.48	-746.5	
**2**	Canada	0-14	female	-0.13	-0.21	6.18	4.79	4.63	
**2**	Canada	0-14	male	-0.45	-0.96	2.27	3.12	0.19	
**2**	Canada	15-64	female	11.03	15.84	19.35	12.77	2.61	
**2**	Canada	15-64	male	22.21	37.22	28.93	16.49	-8.35	7.97
**2**	Canada	65-74	female	23.98	63.79	113.11	74.85	38.39	
**2**	Canada	65-74	male	50.01	113.05	161.44	62.1	-10.56	2.73
**2**	Canada	75-84	female	115.01	116.24	333.16	232.48	128.71	
**2**	Canada	75-84	male	215.85	259.46	530.44	305.3	227.55	
**2**	Canada	85over	female	401.05	-272.18	744.83	-32.71	-602.84	72.74
**2**	Canada	85over	male	532.49	-101.75	1243.94	15.16	-610.52	36.13
**2**	Switzerland	0-14	female	1.33	-0.58	2.99	-1.37	1.1	
**2**	Switzerland	0-14	male	6.61	4.06	7	5.74	2.95	
**2**	Switzerland	15-64	female	0.51	4.05	3.93	-0.76	2.9	
**2**	Switzerland	15-64	male	10.9	18.73	16.34	8.26	9.38	
**2**	Switzerland	65-74	female	23.73	0.59	23.24	-18.97	-16.85	75.31
**2**	Switzerland	65-74	male	126.57	122.21	68.17	0.16	1.37	
**2**	Switzerland	75-84	female	229.76	137.99	249.35	169.98	195.74	
**2**	Switzerland	75-84	male	605.17	250.86	481.26	372.37	369.57	
**2**	Switzerland	85over	female	1711.32	414.69	1358.98	857.29	575.35	
**2**	Switzerland	85over	male	2885.67	842.53	1997.69	1147.75	964.05	
**2**	Germany - Total National Population	0-14	female	-0.68	-0.15	2.57	2.08	3.23	
**2**	Germany - Total National Population	0-14	male	-4.09	-2.31	-0.76	-0.68	0.49	
**2**	Germany - Total National Population	15-64	female	-0.61	8.97	12.75	8.99	7.24	
**2**	Germany - Total National Population	15-64	male	10.49	35.13	33.38	27.65	19.4	

© 2026 Chen X et al. *JAMA Network Open*.

**2**	Germany - Total National Population	65-74	female	6.36	98.61	136.39	126.4	93.47	
**2**	Germany - Total National Population	65-74	male	65.52	243.81	269.81	197.93	137.31	
**2**	Germany - Total National Population	75-84	female	136.92	335.41	503.02	358.47	220.77	
**2**	Germany - Total National Population	75-84	male	246.02	500.38	704.66	438.63	157.77	
**2**	Germany - Total National Population	85over	female	72.58	35.31	732.96	-464.73	-1112	187.52
**2**	Germany - Total National Population	85over	male	422.22	481.97	1161.72	-157.82	-848.97	48.73
**2**	Finland	0-14	female	-5.37	1.09	-0.03	0.78	1.94	
**2**	Finland	0-14	male	-1.68	-2.57	0.68	-2.07	-2.19	
**2**	Finland	15-64	female	-5.49	-0.93	5.01	-0.58	-7.11	
**2**	Finland	15-64	male	18.75	15.01	20.79	21.58	8.68	
**2**	Finland	65-74	female	29.94	16.54	132.28	64.35	-26.07	10.72
**2**	Finland	65-74	male	-1.51	127.08	193.93	99.68	56.35	
**2**	Finland	75-84	female	43.3	161.72	377.46	300.95	141.82	
**2**	Finland	75-84	male	38.26	0.31	536.84	339.77	-13.28	1.45
**2**	Finland	85over	female	24.21	448.05	2040.47	1085.66	82.86	
**2**	Finland	85over	male	-301.16	778.2	2442.19	1757.32	176.23	
**2**	France - Total National Population	0-14	female	-1.4	-0.76	2.75	2.09	2.18	
**2**	France - Total National Population	0-14	male	-3.36	-2.02	0.24	-2.67	-0.39	
**2**	France - Total National Population	15-64	female	0.63	-5.41	-4.68	-8.69	-10.74	
**2**	France - Total National Population	15-64	male	10.66	8.4	10.87	1.05	2.27	
**2**	France - Total National Population	65-74	female	41.03	37.29	37.71	-13.67	-29.65	37.34
**2**	France - Total National Population	65-74	male	139.58	124.58	90.07	-11.74	-48.69	17.06
**2**	France - Total National Population	75-84	female	224.94	79.62	83.81	-66.62	-89.45	40.18
**2**	France - Total National Population	75-84	male	519.74	384.55	290.83	80.03	42.25	
**2**	France - Total National Population	85over	female	945.62	732.88	1272.22	470.4	507.53	
**2**	France - Total National Population	85over	male	1562.11	1559.46	1949.34	641.6	707.97	
**2**	Netherlands	0-14	female	1.36	-0.05	-0.41	-0.15	-0.2	38.98
**2**	Netherlands	0-14	male	0.58	-1.8	-1.99	-0.48	-2.22	
**2**	Netherlands	15-64	female	1.52	15.69	11.39	7.69	13.11	
**2**	Netherlands	15-64	male	11.75	22.89	18.22	15.05	15.76	
**2**	Netherlands	65-74	female	55.72	88.42	62.89	47.82	51.33	
**2**	Netherlands	65-74	male	164.16	209.5	127.21	118.16	94.1	
**2**	Netherlands	75-84	female	292.55	335.87	273.43	256.48	244.74	
**2**	Netherlands	75-84	male	687.09	742.79	435.4	392.74	383.16	
**2**	Netherlands	85over	female	1064.75	805.75	1157.26	782.58	852.14	
**2**	Netherlands	85over	male	1842.79	1546.37	890.9	495.96	451.8	
**2**	Sweden	0-14	female	0.73	-0.34	2.84	1.84	2.69	
**2**	Sweden	0-14	male	5.43	2.72	4.74	5.69	7	
**2**	Sweden	15-64	female	0.83	2.77	3.51	6.71	3.14	
**2**	Sweden	15-64	male	15.35	17.91	14.24	16.33	11.13	
**2**	Sweden	65-74	female	18.97	-1	1.49	-29.62	-80.24	564.36
**2**	Sweden	65-74	male	116.03	80.28	35.23	26.21	-68.54	26.59
**2**	Sweden	75-84	female	278.66	122.92	250.94	245.1	195.33	
**2**	Sweden	75-84	male	486.85	179.14	315.22	334.09	218.53	
**2**	Sweden	85over	female	1038.37	-303.23	266.02	69.37	-254.89	23.81
**2**	Sweden	85over	male	1879.43	145.74	635.05	342.78	-556.87	18.54
**3**	Belgium	0-14	female	-0.27	-3.41	-2.55	-9.41	-8.6	
**3**	Belgium	0-14	male	-7.86	-5.23	-7.09	-13.05	-14.17	
**3**	Belgium	15-64	female	5.35	4.88	8.6	3.43	3.46	
**3**	Belgium	15-64	male	25.02	24.57	19.38	15.93	18.71	

© 2026 Chen X et al. *JAMA Network Open*.

**3**	Belgium	65-74	female	85.66	48.89	31.22	-24.46	-43.05	40.72
**3**	Belgium	65-74	male	263.28	163.47	115.09	-19.76	-3.09	4.22
**3**	Belgium	75-84	female	622.85	170.53	277.77	114.64	105.31	
**3**	Belgium	75-84	male	918.3	391.41	208.55	54.02	59.89	
**3**	Belgium	85over	female	2686.85	-187.04	1378.1	643	781.56	
**3**	Belgium	85over	male	2994.72	380.37	1225.91	366.86	115.21	
**3**	Spain	0-14	female	-1.64	0.08	0.66	1.95	3	
**3**	Spain	0-14	male	-2.27	-1.43	2.87	0.69	4.47	
**3**	Spain	15-64	female	9.09	5.28	4.89	-1.49	-4.19	29.5
**3**	Spain	15-64	male	18.94	17.44	10.32	-4.92	-7.96	27.57
**3**	Spain	65-74	female	109.09	72.11	69.68	36.02	35.38	
**3**	Spain	65-74	male	244.25	163.02	112.05	9.82	-34.88	6.59
**3**	Spain	75-84	female	501.54	198.43	224.68	82.39	104.2	
**3**	Spain	75-84	male	883.75	401.23	385.11	203.36	252.08	
**3**	Spain	85over	female	2196.77	626.75	1609.02	839.16	1275.8	
**3**	Spain	85over	male	2726.5	1044.52	1887.66	790.94	1205.28	
**3**	England and Wales	0-14	female	-2.73	-0.2	-0.02	1.46	2.51	
**3**	England and Wales	0-14	male	-1.16	0.7	3.34	6.56	6.46	
**3**	England and Wales	15-64	female	15.29	22.03	12.89	13.27	8.53	
**3**	England and Wales	15-64	male	30.26	42.2	17.44	21.93	9.65	
**3**	England and Wales	65-74	female	115.05	127.27	75.34	61.58	4.76	
**3**	England and Wales	65-74	male	230.02	200.92	100.27	77.57	-3.79	0.62
**3**	England and Wales	75-84	female	475.59	302.34	244.67	242.76	122.2	
**3**	England and Wales	75-84	male	804.76	475.24	277.96	290.09	121.61	
**3**	England and Wales	85over	female	1823.75	928.4	988.05	1057.55	563.11	
**3**	England and Wales	85over	male	2379.29	1125.01	1353.78	1329.16	831.19	
**3**	Northern Ireland	0-14	female	-4.73	3.88	0.67	-8.13	0.1	
**3**	Northern Ireland	0-14	male	-4.42	4.8	3.81	2.88	5.76	
**3**	Northern Ireland	15-64	female	12.65	19.4	-5.03	8.67	11.44	
**3**	Northern Ireland	15-64	male	25.52	36.6	20.68	-0.43	47.87	
**3**	Northern Ireland	65-74	female	168.93	197.61	154	161.48	191.79	
**3**	Northern Ireland	65-74	male	234.9	265.56	271.35	241.42	447.4	
**3**	Northern Ireland	75-84	female	379.87	311.09	214.87	233.93	278.4	
**3**	Northern Ireland	75-84	male	660.91	781.96	450.99	628.07	613.6	
**3**	Northern Ireland	85over	female	1301.5	775.88	1293.24	1200.2	890.52	
**3**	Northern Ireland	85over	male	1195.25	671.84	169.42	271.01	453.37	
**3**	Scotland	0-14	female	-2.07	3.67	-0.64	0.79	-0.35	20.28
**3**	Scotland	0-14	male	-1.62	2.71	2.55	10.72	5.62	
**3**	Scotland	15-64	female	0.61	18.98	-2.91	-3.55	-15.28	112.89
**3**	Scotland	15-64	male	37.79	37.55	0.24	1.11	-10.54	13.74
**3**	Scotland	65-74	female	96.77	145.12	134.08	109.36	50.3	
**3**	Scotland	65-74	male	279.68	363.48	304.76	320.78	262.93	
**3**	Scotland	75-84	female	367.6	333.39	184.63	220.03	54.11	
**3**	Scotland	75-84	male	604.38	386.81	224.1	153.02	-78.8	5.76
**3**	Scotland	85over	female	1543.11	944.25	1432.99	1391.28	881.86	
**3**	Scotland	85over	male	2113.13	1726.31	2188.88	2522.53	2296.54	
**3**	Portugal	0-14	female	-4.26	-6.75	-4.07	-7.81	-9.09	
**3**	Portugal	0-14	male	-5.52	-3.04	-1.08	1.84	0.2	
**3**	Portugal	15-64	female	1.63	7.43	3.4	-3.38	-7.7	88.91
**3**	Portugal	15-64	male	24.16	23.02	14.91	0.91	-8.13	12.9

© 2026 Chen X et al. *JAMA Network Open*.

**3**	Portugal	65-74	female	78.94	100.46	78.95	58.46	53.61		
**3**	Portugal	65-74	male	111.97	201.81	115.24	59.41	41.51		
**3**	Portugal	75-84	female	337.48	320.39	250.26	3.16	-80.05		8.78
**3**	Portugal	75-84	male	321.62	320.66	221.43	-124.86	-238.75		42.1
**3**	Portugal	85over	female	1243.37	1149.51	1575.21	560.06	620.46		
**3**	Portugal	85over	male	1266.83	1537.19	1332.89	411.02	410.41		
**3**	Slovenia	0-14	female	-1.09	2.53	-0.3	-5.03	2.65		
**3**	Slovenia	0-14	male	-3.07	-6.27	4.63	-1.49	-2.67		
**3**	Slovenia	15-64	female	-6.12	1.06	6.46	-15.84	-9.05		1783.42
**3**	Slovenia	15-64	male	14.14	52.43	27.71	34.62	17.57		
**3**	Slovenia	65-74	female	69.83	153.65	73.18	51.44	36.16		
**3**	Slovenia	65-74	male	207.44	345.13	159.52	40.63	-46.42		6.17
**3**	Slovenia	75-84	female	742.96	496.01	368.94	220.52	75.66		
**3**	Slovenia	75-84	male	1001.75	935.01	494.17	383.6	127.41		
**3**	Slovenia	85over	female	2562.27	521.72	753.78	-548.38	-295.2		21.98
**3**	Slovenia	85over	male	2946.68	842.76	-4	-812.37	-656.49		38.8
**4**	Austria	0-14	female	1.75	0.61	0.24	-2.13	2.45		
**4**	Austria	0-14	male	1.15	-4.35	-5.49	-0.36	-2.1		
**4**	Austria	15-64	female	3.06	7.2	9.05	2.8	3.65		
**4**	Austria	15-64	male	16.94	39.24	33.33	23.76	24.21		
**4**	Austria	65-74	female	37.87	101.11	49.44	-3.87	-1.96		3.09
**4**	Austria	65-74	male	168.64	249.84	252.07	126.79	87.15		
**4**	Austria	75-84	female	464.27	597.79	784.04	733.17	749.38		
**4**	Austria	75-84	male	802.76	919.45	1032.18	977.88	843.49		
**4**	Austria	85over	female	1091.24	644.87	1199.66	-13.72	-822.59		28.49
**4**	Austria	85over	male	1649.68	1157.58	1825.73	530.7	-900.39		17.44
**4**	Czechia	0-14	female	-5.51	-4.81	-2.58	-5.01	-5.37		
**4**	Czechia	0-14	male	-2.79	-2.07	-4.73	-6.08	-5.1		
**4**	Czechia	15-64	female	8.45	37.54	3.76	-8.4	-10.35		37.7
**4**	Czechia	15-64	male	25.01	91.25	17.08	7.27	9.45		
**4**	Czechia	65-74	female	127.68	419.54	51.01	1	-38.36		6.4
**4**	Czechia	65-74	male	366.6	887.18	148.62	-55.78	-78.97		9.61
**4**	Czechia	75-84	female	742.46	1141.62	616.64	361.87	444.39		
**4**	Czechia	75-84	male	1361.12	2136.33	693.99	368.36	407.67		
**4**	Czechia	85over	female	2236.12	2213.24	1404.37	135.38	235.85		
**4**	Czechia	85over	male	3520.61	4070.8	2454.89	1199.53	856.59		
**4**	Estonia	0-14	female	2.76	2.88	9.42	12.04	6.76		
**4**	Estonia	0-14	male	-6.62	8.79	4.64	5	0.74		
**4**	Estonia	15-64	female	21.54	42.13	33.17	38.72	43.24		
**4**	Estonia	15-64	male	28.94	96.49	83.23	67.22	86.67		
**4**	Estonia	65-74	female	7.59	350.86	116.89	34.71	16.69		
**4**	Estonia	65-74	male	-20.18	576.69	222.14	-76.95	-99.96		22.72
**4**	Estonia	75-84	female	0.75	654.53	303.77	-93.89	-167.78		27.28
**4**	Estonia	75-84	male	184.92	1658.36	1045.94	415.36	295.43		
**4**	Estonia	85over	female	79.33	2120.52	1027.32	-871.75	-866.47		53.86
**4**	Estonia	85over	male	-440.44	2631.71	544.29	-978.28	-2826.25		139.08
**4**	Greece	0-14	female	-5.75	-1.84	-3.25	-3.98	-0.35		
**4**	Greece	0-14	male	0.61	4.03	-0.5	3.05	8.19		
**4**	Greece	15-64	female	9.09	31.88	12.7	3.71	-0.69		1.2
**4**	Greece	15-64	male	5.55	62.92	26.37	3.21	-13.43		13.7
**4**	Greece	65-74	female	51.17	224.74	78.71	-21.84	-37.28		16.67

© 2026 Chen X et al. *JAMA Network Open*.

**4**	Greece	65-74	male	160.21	412.11	190.51	25.97	21.05	
**4**	Greece	75-84	female	239.51	519.31	439.96	110.57	-3.25	0.25
**4**	Greece	75-84	male	210.68	729.8	608.17	173.05	35.42	
**4**	Greece	85over	female	558.05	1321.01	1468.45	-607.27	-933.96	46.04
**4**	Greece	85over	male	760.4	1937.13	1813.52	2.2	-504.21	11.17
**4**	Hungary	0-14	female	-0.82	0.54	7.2	1.7	9.05	
**4**	Hungary	0-14	male	0.1	0.8	2.37	3.33	5.65	
**4**	Hungary	15-64	female	14.47	80.85	8.8	-1.53	-0.88	2.31
**4**	Hungary	15-64	male	18	125.59	10.63	-31.05	-34.25	42.34
**4**	Hungary	65-74	female	209.54	531.61	201.56	140.56	146.41	
**4**	Hungary	65-74	male	245.06	918.59	210.14	71.07	-51.69	3.58
**4**	Hungary	75-84	female	470.63	1025.12	373.78	-2.43	26.97	
**4**	Hungary	75-84	male	934.93	1856.02	842.54	485.1	519.07	
**4**	Hungary	85over	female	964.9	1469.36	699.33	-314.3	-379.74	22.15
**4**	Hungary	85over	male	1540.55	2484.97	1362.57	8.91	-8.83	0.16
**4**	Italy	0-14	female	-1.69	-0.26	-2.34	0.42	0.74	
**4**	Italy	0-14	male	-1.81	-2.13	-1.71	0.65	1.04	
**4**	Italy	15-64	female	8.83	13.7	8.52	4.79	1.31	
**4**	Italy	15-64	male	27.15	34.06	21.56	12.95	11.45	
**4**	Italy	65-74	female	118.16	144.54	107.15	49.5	38.32	
**4**	Italy	65-74	male	351.45	295.29	174.41	85.44	22.53	
**4**	Italy	75-84	female	521.8	434.04	445.11	255.13	157.46	
**4**	Italy	75-84	male	1004.45	676.4	562.78	199.84	69.14	
**4**	Italy	85over	female	1959.27	1021.24	1904.54	555.61	294.49	
**4**	Italy	85over	male	2320.41	1361.21	2037.89	524.46	305.75	
**4**	United States of America (USA)	0-14	female	-1.26	2.49	5.45	5.62	7.06	
**4**	United States of America (USA)	0-14	male	-1.5	0.86	5.16	5.74	5.56	
**4**	United States of America (USA)	15-64	female	36.51	71.33	31.71	5.6	-8.21	5.65
**4**	United States of America (USA)	15-64	male	74.9	127.87	56.43	13.98	-20.22	7.4
**4**	United States of America (USA)	65-74	female	226.21	330.35	197.17	86.61	71.28	
**4**	United States of America (USA)	65-74	male	355.5	467.91	228.31	43.35	-4.23	0.39
**4**	United States of America (USA)	75-84	female	673.58	726.35	523.9	261.9	239.31	
**4**	United States of America (USA)	75-84	male	976.43	999.51	614.52	176.09	131.08	
**4**	United States of America (USA)	85over	female	1932.3	862.7	822.04	-100.01	-275.35	10.38
**4**	United States of America (USA)	85over	male	2230.41	1474.61	1154.4	13.58	-116.78	2.4
**5**	Bulgaria	0-14	female	-6.13	4.54	-4.9	-3.68	-9.37	
**5**	Bulgaria	0-14	male	-7.3	1.26	-0.5	6.86	2.61	
**5**	Bulgaria	15-64	female	42.47	148.04	21.2	-21.31	-6.74	13.25
**5**	Bulgaria	15-64	male	78.09	230.44	35.65	-27.74	-34.27	18.02
**5**	Bulgaria	65-74	female	336.32	1144.7	325.18	-16.81	13.25	
**5**	Bulgaria	65-74	male	760.83	1849.08	582.32	-55.74	-85.76	4.43
**5**	Bulgaria	75-84	female	642.7	2322.08	849.18	78.72	290.65	
**5**	Bulgaria	75-84	male	1629.68	3971.83	1667.47	161.13	247.14	
**5**	Bulgaria	85over	female	1584.1	4653.04	3028.61	1221.3	1972.3	
**5**	Bulgaria	85over	male	3411.5	7179.63	4919.87	684.23	1514.18	
**5**	Croatia	0-14	female	3.76	7.72	3.28	6.04	6.27	
**5**	Croatia	0-14	male	-3.25	-4.45	1.13	0.33	-11.22	
**5**	Croatia	15-64	female	9.56	42.76	3.26	-3.69	-7.52	20.16
**5**	Croatia	15-64	male	11.58	64.33	13.51	1.32	-22.01	24.25
**5**	Croatia	65-74	female	193.83	492.92	314.3	206.86	220.93	
**5**	Croatia	65-74	male	390.82	843.54	397.28	239.08	169.61	

© 2026 Chen X et al. *JAMA Network Open*.

**5**	Croatia	75-84	female	550.96	1099.97	651.97	-1.01	-60.88	2.69
**5**	Croatia	75-84	male	1016.72	2151.87	1207.17	473.5	615.86	
**5**	Croatia	85over	female	1495.44	3180.69	2545.35	266.4	1211.47	
**5**	Croatia	85over	male	1647.24	3867.9	2522.88	-72.12	583.82	
**5**	Lithuania	0-14	female	-0.18	4.86	5.06	7.37	9.47	
**5**	Lithuania	0-14	male	-0.06	5.48	-2.15	4.75	-4.41	54.98
**5**	Lithuania	15-64	female	40.29	91.19	45.5	24.83	30.81	
**5**	Lithuania	15-64	male	121.49	188.41	110.26	103.17	125.73	
**5**	Lithuania	65-74	female	225.14	490.71	262.94	100.87	156.98	
**5**	Lithuania	65-74	male	739.38	1144.94	747.01	302.95	508.73	
**5**	Lithuania	75-84	female	623.1	1510.85	785.98	310.32	271.79	
**5**	Lithuania	75-84	male	1254.11	2086.4	1395.47	710.48	473.21	
**5**	Lithuania	85over	female	1401.63	3264.66	2529.48	237.43	341.92	
**5**	Lithuania	85over	male	1675.23	4995.08	2449.48	130.25	114.01	
**5**	Latvia	0-14	female	-0.19	-8.14	2.16	-0.54	-6.95	
**5**	Latvia	0-14	male	7.49	3.73	2.89	1.17	1.1	
**5**	Latvia	15-64	female	-4.83	73.01	9.17	-21.92	-45.55	87.22
**5**	Latvia	15-64	male	-8.12	192.84	133.82	79.11	3.72	
**5**	Latvia	65-74	female	-4.88	492.01	257.12	156.85	-4.43	0.49
**5**	Latvia	65-74	male	159.3	1098.9	474.75	236.85	-140.89	7.15
**5**	Latvia	75-84	female	449.17	1706.38	873.95	333.73	210.17	
**5**	Latvia	75-84	male	17.53	1553	236.94	-821.41	-1198.67	111.76
**5**	Latvia	85over	female	267.12	2475.25	528.81	-1610.92	-2565.98	127.69
**5**	Latvia	85over	male	1037.21	3376.52	998.88	-1193.42	-2100.2	60.85
**5**	Poland	0-14	female	-5.58	-2.81	-2.66	-4.98	-9	
**5**	Poland	0-14	male	-3.01	0.2	1.1	-1.3	-2.56	
**5**	Poland	15-64	female	6.45	44.53	3.86	-16.49	-26.03	77.52
**5**	Poland	15-64	male	33.76	96.95	14.42	-38.81	-55.17	64.75
**5**	Poland	65-74	female	207.55	467.4	86.53	-46.59	-104.33	19.82
**5**	Poland	65-74	male	604.52	957.2	254.18	-87.2	-162.67	13.76
**5**	Poland	75-84	female	546.13	1246.38	364.42	-130.46	-269.09	18.52
**5**	Poland	75-84	male	1424.07	2065.01	491.59	-258.79	-484.82	18.68
**5**	Poland	85over	female	2315.82	3115.53	1566.32	-240.69	-437.25	9.69
**5**	Poland	85over	male	4480.69	4869.99	2193.47	137.06	-140.81	1.21
**5**	Slovakia	0-14	female	-0.43	-2.39	0.14	-2.68	-5.15	
**5**	Slovakia	0-14	male	1.03	-2.41	2.21	2.19	0.1	
**5**	Slovakia	15-64	female	5.82	72.93	7.03	-4.67	-9.51	16.53
**5**	Slovakia	15-64	male	15.5	155.33	40.96	1.03	-3.03	1.42
**5**	Slovakia	65-74	female	147.31	733.12	141.64	80.45	23.92	
**5**	Slovakia	65-74	male	281.33	1271.47	321.76	-31.85	-74.92	5.7
**5**	Slovakia	75-84	female	614.75	2046.24	723.33	198.6	150.95	
**5**	Slovakia	75-84	male	1145.23	2978.05	990.41	305.04	218.12	
**5**	Slovakia	85over	female	955.82	3443.79	1461.63	-621.29	-476.97	18.74
**5**	Slovakia	85over	male	1751.71	4155.56	1479.83	-627.37	-482.11	15.02

Note:
a. Excess deaths per 100k people are shown above;
b. Harvesting effect ratio was defined as the proportion of cumulative excess mortality in 2020-2022 or 2020-2023 that was offset by cumulative negative excess mortality during 2023 and 2024 combined or 2024 alone;
c. A harvesting percentage exceeding 100% indicates that the subsequent mortality deficit entirely offset or surpassed the initial mortality surge, while missing values indicate that the required pattern of all-positive surge years followed by all-negative deficit years was not observed in that group, and therefore no harvesting effect was computed.
d. The harvesting effect ratio was calculated based on Eq. #(4) and #(5) in the main text; the same procedure for eTable 1 described in the main text was applied here, with the only difference being that the calculations were further stratified by sex.

© 2026 Chen X et al. *JAMA Network Open*.

eTable 3. The Linear Trend in Mortality Rates During 2015 to 2019 by Quintile and Country

quintile	country	rate (95% CI)	p-value	trend
1	Australia	-0.0212 (-0.0234 to -0.0190)	p<0.001	Negative
1	Denmark	-0.0016 (-0.0053 to 0.0020)	0.379	No change
1	Iceland	-0.0358 (-0.0497 to -0.0219)	p<0.001	Negative
1	Israel	-0.0287 (-0.0331 to -0.0243)	p<0.001	Negative
1	Luxembourg	-0.0181 (-0.0289 to -0.0072)	0.001	Negative
1	Norway	-0.0149 (-0.0191 to -0.0107)	p<0.001	Negative
1	New Zealand	-0.0182 (-0.0223 to -0.0142)	p<0.001	Negative
2	Canada	-0.0039 (-0.0069 to -0.0009)	0.012	Negative
2	Switzerland	-0.0138 (-0.0195 to -0.0080)	p<0.001	Negative
2	Germany	-0.0004 (-0.0053 to 0.0045)	0.863	No change
2	Finland	0.0031 (-0.0008 to 0.0070)	0.116	No change
2	France	0.0025 (-0.0015 to 0.0065)	0.222	No change
2	Netherlands	-0.0026 (-0.0066 to 0.0014)	0.202	No change
2	Sweden	-0.0315 (-0.0353 to -0.0277)	p<0.001	Negative
3	Belgium	-0.0101 (-0.0152 to -0.0050)	p<0.001	Negative
3	Spain	-0.0051 (-0.0100 to -0.0002)	0.04	Negative
3	England & Wales	-0.0105 (-0.0183 to -0.0027)	0.008	Negative
3	Northern Ireland	-0.0073 (-0.0173 to 0.0026)	0.149	No change
3	Scotland	-0.0026 (-0.0098 to 0.0046)	0.482	No change
3	Portugal	0.0096 (0.0033 to 0.0160)	0.003	Positive
3	Slovenia	0.0061 (0.0001 to 0.0122)	0.047	Positive
4	Austria	-0.0092 (-0.0140 to -0.0043)	0	Negative
4	Czechia	0.0052 (0.0002 to 0.0102)	0.04	Positive
4	Estonia	-0.0007 (-0.0069 to 0.0055)	0.818	No change
4	Greece	0.0127 (0.0074 to 0.0181)	p<0.001	Positive
4	Hungary	0.0037 (-0.0021 to 0.0095)	0.21	No change
4	Italy	0.0028 (-0.0021 to 0.0077)	0.259	No change
4	United States of America	0.0013 (-0.0007 to 0.0033)	0.205	No change
5	Bulgaria	0.0115 (0.0063 to 0.0167)	p<0.001	Positive
5	Croatia	0.0106 (0.0046 to 0.0167)	0.001	Positive
5	Lithuania	-0.0006 (-0.0060 to 0.0049)	0.834	No change
5	Latvia	0.0120 (0.0068 to 0.0172)	p<0.001	Positive
5	Poland	0.0145 (0.0103 to 0.0187)	p<0.001	Positive
5	Slovakia	-0.0011 (-0.0065 to 0.0042)	0.679	No change

Note: Trends are classified based on statistical significance. Countries with significant p-values ($p < 0.05$) are categorized as "Positive" or "Negative".

© 2026 Chen X et al. *JAMA Network Open*.

eTable 4. Annual Excess Death Rate per 100,000 Population and Estimated Harvesting Effect, by Country, 2020-2024, Relative to Expected Deaths Based on 2015-2019 Mortality (the Sensitivity Analysis Results for the Negative Binomial Model)

Quintile	Country	2020	2021	2022	2023	2024	Harvesting effect ratio (%)[d,e,f]
1	Australia	-16 (-21, -10)[a]	14 (10, 19)[a]	76 (63, 89)[a]	35 (27, 43)[a]	36 (29, 43)[a]	.
1	Denmark	-21 (-31, -11)[a]	12 (-2, 25)[c]	34 (23, 46)[a]	-3 (-14, 8)	-44 (-58, -30)[a]	185 (-181, 552)
1	Iceland	3 (-22, 29)	9 (-17, 36)	99 (66, 132)[a]	58 (29, 88)[a]	60 (30, 91)[a]	.
1	Israel	20 (13, 27)[a]	32 (24, 39)[a]	31 (23, 39)[a]	-2 (-9, 4)	8 (4, 13)[a]	.
1	Luxembourg	24 (0, 47)[b]	5 (-15, 24)	-17 (-36, 2)[c]	-42 (-63, -21)[a]	-46 (-65, -27)[a]	746 (-6197, 7688)
1	Norway	-10 (-18, -1)[b]	14 (1, 26)[b]	77 (61, 92)[a]	34 (24, 44)[a]	35 (25, 44)[a]	.
1	New Zealand	-31 (-38, -23)[a]	-10 (-16, -4)[a]	26 (19, 34)[a]	4 (-3, 11)	-14 (-19, -8)[a]	.
2	Canada	32 (23, 40)[a]	28 (22, 34)[a]	70 (59, 80)[a]	28 (23, 34)[a]	-6 (-14, 2)	4 (-1, 9)
2	Switzerland	93 (63, 123)[a]	40 (26, 54)[a]	77 (62, 93)[a]	44 (32, 56)[a]	40 (30, 51)[a]	.
2	Germany	27 (13, 41)[a]	70 (51, 89)[a]	112 (90, 134)[a]	47 (33, 60)[a]	-2 (-17, 12)	1 (-3, 5)
2	Finland	6 (-4, 17)	35 (22, 49)[a]	125 (103, 147)[a]	80 (61, 99)[a]	11 (1, 22)[b]	.
2	France	73 (51, 95)[a]	56 (44, 69)[a]	71 (56, 86)[a]	14 (6, 21)[a]	10 (2, 18)[b]	.
2	Netherlands	76 (52, 99)[a]	86 (68, 104)[a]	67 (54, 80)[a]	54 (43, 65)[a]	56 (46, 65)[a]	.
2	Sweden	72 (52, 93)[a]	17 (6, 28)[a]	38 (27, 49)[a]	32 (20, 44)[a]	3 (-6, 11)	.
3	Austria	91 (66, 116)[a]	103 (85, 121)[a]	125 (102, 148)[a]	78 (61, 95)[a]	47 (32, 62)[a]	.
3	Belgium	152 (113, 192)[a]	36 (23, 49)[a]	69 (53, 85)[a]	23 (14, 33)[a]	24 (15, 33)[a]	.
3	Spain	146 (101, 191)[a]	62 (47, 77)[a]	90 (70, 109)[a]	35 (26, 45)[a]	47 (34, 61)[a]	.
3	England and Wales	118 (84, 153)[a]	84 (62, 106)[a]	62 (47, 76)[a]	64 (48, 79)[a]	30 (19, 40)[a]	.
3	Northern Ireland	85 (61, 108)[a]	86 (63, 109)[a]	64 (43, 84)[a]	66 (43, 89)[a]	93 (72, 115)[a]	.
3	Scotland	103 (70, 136)[a]	98 (78, 118)[a]	78 (57, 98)[a]	80 (59, 101)[a]	43 (28, 58)[a]	.
3	Portugal	85 (64, 106)[a]	94 (59, 129)[a]	86 (65, 107)[a]	16 (5, 28)[a]	4 (-11, 19)	.
4	Czechia	150 (111, 190)[a]	269 (222, 315)[a]	93 (75, 111)[a]	30 (18, 41)[a]	31 (20, 43)[a]	.
4	Estonia	19 (-1, 39)[c]	195 (163, 227)[a]	109 (87, 131)[a]	15 (-8, 37)	-1 (-22, 21)	0 (-4, 5)
4	Greece	55 (40, 71)[a]	170 (139, 200)[a]	127 (103, 151)[a]	-3 (-15, 9)	-36 (-53, -20)[a]	11 (6, 17)[a]
4	Hungary	101 (69, 133)[a]	272 (228, 315)[a]	87 (70, 103)[a]	12 (-1, 24)[c]	7 (-9, 23)	.
4	Italy	175 (135, 215)[a]	128 (108, 148)[a]	142 (117, 168)[a]	56 (44, 67)[a]	32 (20, 44)[a]	.
4	Slovenia	146 (103, 189)[a]	108 (81, 134)[a]	67 (49, 86)[a]	20 (2, 37)[b]	2 (-16, 19)	.
4	United States of America (USA)	141 (121, 160)[a]	166 (147, 185)[a]	97 (85, 110)[a]	25 (21, 29)[a]	2 (-2, 7)	.
5	Bulgaria	220 (159, 282)[a]	625 (537, 712)[a]	228 (183, 273)[a]	10 (-3, 23)	38 (21, 55)[a]	.
5	Croatia	128 (89, 167)[a]	300 (252, 348)[a]	173 (141, 204)[a]	45 (30, 61)[a]	57 (37, 76)[a]	.
5	Lithuania	180 (141, 220)[a]	358 (304, 411)[a]	221 (185, 257)[a]	90 (71, 109)[a]	106 (88, 125)[a]	.
5	Latvia	38 (13, 63)[a]	354 (302, 406)[a]	150 (123, 178)[a]	-4 (-31, 23)	-108 (-136, -81)[a]	21 (14, 28)[a]
5	Poland	160 (119, 201)[a]	279 (236, 321)[a]	83 (67, 99)[a]	-38 (-47, -29)[a]	-70 (-80, -60)[a]	21 (17, 24)[a]
5	Slovakia	86 (62, 111)[a]	357 (304, 409)[a]	107 (88, 127)[a]	6 (-5, 17)	-4 (-16, 8)	1 (-1, 2)

Note:
a. $p<0.01$, b. $p<0.05$, c. $p<0.1$, d. Excess deaths per 100k people.
e. Harvesting effect ratio was defined as the proportion of cumulative excess mortality in 2020-2022 or 2020-2023 that was offset by cumulative negative excess mortality during 2023 and 2024 combined or 2024 alone;
f. A harvesting percentage exceeding 100% indicates that the subsequent mortality deficit entirely offset or surpassed the initial mortality surge, while missing values suggest that the required pattern of all-positive surge years followed by all-negative deficit years was not observed in that group. Therefore, no harvesting effect was computed.

© 2026 Chen X et al. *JAMA Network Open*.

eTable 5. Annual Excess Death Rate per 100,000 Population and Estimated Harvesting Effect, by Country, 2020-2024, Relative to Expected Deaths Based on 2015-2019 Mortality (the Sensitivity Analysis Results for the Broader Age Band)

Quintile	Country	2020	2021	2022	2023	2024	Harvesting effect ratio (%)[d,e,f]
1	Australia	-16 (-21, -11)[a]	15 (10, 20)[a]	81 (66, 96)[a]	39 (31, 47)[a]	40 (32, 47)[a]	.
1	Denmark	-21 (-31, -10)[a]	12 (-2, 26)[c]	35 (23, 47)[a]	-2 (-13, 8)	-43 (-57, -29)[a]	176 (-1032, 1385)
1	Iceland	3 (-24, 31)	9 (-17, 35)	99 (64, 133)[a]	59 (28, 90)[a]	62 (31, 92)[a]	.
1	Israel	28 (19, 36)[a]	42 (32, 52)[a]	39 (28, 49)[a]	5 (-11, 21)	14 (9, 19)[a]	.
1	Luxembourg	25 (-0, 50)[c]	1 (-20, 21)	-23 (-44, -2)[b]	-46 (-68, -23)[a]	-55 (-75, -34)[a]	3405 (-5671, 12480)
1	Norway	-9 (-18, -1)[b]	14 (3, 26)[b]	77 (61, 94)[a]	35 (24, 46)[a]	36 (25, 47)[a]	.
1	New Zealand	-45 (-56, -34)[a]	-16 (-23, -10)[a]	40 (29, 51)[a]	8 (0, 16)[b]	-22 (-30, -15)[a]	.
2	Canada	33 (25, 42)[a]	32 (26, 38)[a]	77 (65, 88)[a]	34 (28, 40)[a]	-2 (-9, 6)	1 (-2, 4)
2	Switzerland	94 (63, 124)[a]	41 (27, 55)[a]	79 (61, 96)[a]	46 (33, 59)[a]	43 (32, 54)[a]	.
2	Germany	28 (12, 43)[a]	71 (53, 90)[a]	113 (88, 138)[a]	49 (36, 62)[a]	-0 (-14, 14)	0 (-3, 3)
2	Finland	7 (-4, 17)	36 (23, 50)[a]	126 (103, 148)[a]	81 (62, 101)[a]	13 (2, 25)[b]	.
2	France	73 (49, 97)[a]	57 (44, 70)[a]	72 (56, 88)[a]	15 (6, 23)[a]	11 (4, 19)[a]	.
2	Netherlands	76 (52, 99)[a]	86 (67, 106)[a]	67 (53, 81)[a]	54 (43, 66)[a]	56 (45, 67)[a]	.
2	Sweden	73 (53, 93)[a]	18 (8, 29)[a]	40 (29, 52)[a]	36 (24, 47)[a]	6 (-4, 15)	.
3	Austria	91 (66, 117)[a]	104 (82, 125)[a]	126 (101, 151)[a]	79 (61, 97)[a]	48 (33, 63)[a]	.
3	Belgium	153 (108, 197)[a]	36 (23, 50)[a]	70 (53, 88)[a]	25 (14, 35)[a]	26 (16, 36)[a]	.
3	Spain	147 (99, 195)[a]	64 (48, 79)[a]	92 (71, 113)[a]	38 (28, 48)[a]	51 (36, 66)[a]	.
3	England and Wales	120 (84, 155)[a]	86 (63, 109)[a]	64 (49, 80)[a]	67 (51, 83)[a]	33 (22, 45)[a]	.
3	Northern Ireland	85 (60, 110)[a]	87 (63, 111)[a]	64 (43, 86)[a]	67 (43, 90)[a]	95 (72, 118)[a]	.
3	Scotland	103 (71, 135)[a]	99 (77, 121)[a]	79 (57, 101)[a]	82 (60, 104)[a]	45 (29, 62)[a]	.
3	Portugal	87 (65, 108)[a]	96 (62, 131)[a]	89 (66, 111)[a]	20 (8, 32)[a]	8 (-7, 23)	.
4	Czech	151 (109, 193)[a]	270 (221, 318)[a]	95 (75, 115)[a]	32 (19, 45)[a]	34 (22, 46)[a]	.
4	Estonia	19 (-3, 40)[c]	222 (183, 260)[a]	120 (94, 146)[a]	13 (-13, 39)	-3 (-27, 22)	1 (-4, 6)
4	Greece	56 (41, 72)[a]	172 (138, 205)[a]	130 (104, 155)[a]	1 (-12, 13)	-32 (-48, -16)[a]	9 (4, 14)[a]
4	Hungary	100 (64, 136)[a]	271 (223, 318)[a]	86 (68, 104)[a]	11 (-2, 24)	6 (-10, 22)	.
4	Italy	174 (131, 217)[a]	127 (106, 148)[a]	141 (114, 169)[a]	55 (43, 66)[a]	30 (18, 43)[a]	.
4	Slovenia	144 (96, 192)[a]	107 (80, 134)[a]	68 (49, 88)[a]	14 (-4, 32)	-2 (-18, 14)	1 (-3, 4)
4	United States of America (USA)	141 (118, 163)[a]	166 (147, 186)[a]	98 (84, 113)[a]	26 (22, 31)[a]	4 (-1, 8)[c]	.
5	Bulgaria	220 (153, 287)[a]	625 (527, 722)[a]	229 (178, 279)[a]	11 (-2, 25)	40 (23, 58)[a]	.
5	Croatia	128 (90, 166)[a]	300 (249, 351)[a]	173 (139, 207)[a]	45 (29, 61)[a]	57 (37, 76)[a]	.
5	Lithuania	194 (152, 236)[a]	388 (329, 447)[a]	236 (200, 272)[a]	98 (77, 120)[a]	116 (97, 135)[a]	.
5	Latvia	39 (11, 66)[a]	355 (291, 419)[a]	153 (122, 183)[a]	-1 (-28, 26)	-104 (-137, -71)[a]	19 (12, 27)[a]
5	Poland	159 (117, 202)[a]	278 (231, 326)[a]	84 (66, 101)[a]	-37 (-46, -27)[a]	-67 (-77, -58)[a]	20 (16, 23)[a]
5	Slovakia	86 (61, 111)[a]	357 (293, 420)[a]	107 (86, 128)[a]	6 (-6, 17)	-4 (-15, 8)	1 (-1, 2)

Note:
a. p<0.01, b. p<0.05, c. p<0.1, d. Excess deaths per 100k people.
e. Harvesting effect ratio was defined as the proportion of cumulative excess mortality in 2020-2022 or 2020-2023 that was offset by cumulative negative excess mortality during 2023 and 2024 combined or 2024 alone;
f. A harvesting percentage exceeding 100% indicates that the subsequent mortality deficit entirely offset or surpassed the initial mortality surge, while missing values suggest that the required pattern of all-positive surge years followed by all-negative deficit years was not observed in that group. Therefore, no harvesting effect was computed.

© 2026 Chen X et al. *JAMA Network Open*.

eTable 6. Annual Excess Death Rate per 100,000 Population and Estimated Harvesting Effect, by Country, 2020-2024, Relative to Expected Deaths Based on 2015-2019 Mortality (the Sensitivity Analysis Results From the Autocorrelation Control)

Quintile	Country	2020	2021	2022	2023	2024	Harvesting effect ratio (%)[d,e,f]
1	Australia	-15 (-21, -10)[a]	15 (10, 20)[a]	81 (67, 95)[a]	39 (31, 46)[a]	40 (33, 47)[a]	.
1	Denmark	-21 (-32, -10)[a]	12 (-2, 26)[c]	35 (23, 46)[a]	-2 (-13, 9)	-43 (-57, -30)[a]	174 (-9142, 9491)
1	Iceland	3 (-23, 30)	9 (-17, 35)	99 (65, 132)[a]	58 (29, 87)[a]	60 (28, 92)[a]	.
1	Israel	27 (19, 36)[a]	42 (33, 50)[a]	38 (28, 48)[a]	4 (-11, 19)	13 (8, 18)[a]	.
1	Luxembourg	25 (-1, 50)[c]	1 (-20, 22)	-23 (-45, -0)[b]	-45 (-67, -24)[a]	-54 (-75, -34)[a]	2915 (-3975, 9806)
1	Norway	-9 (-17, -2)[b]	14 (2, 26)[b]	77 (62, 92)[a]	34 (23, 45)[a]	35 (24, 46)[a]	.
1	New Zealand	-45 (-55, -35)[a]	-16 (-22, -9)[a]	41 (31, 51)[a]	9 (1, 17)[b]	-21 (-28, -14)[a]	.
2	Canada	33 (25, 41)[a]	32 (26, 38)[a]	76 (66, 86)[a]	33 (27, 38)[a]	-3 (-11, 5)	2 (-2, 6)
2	Switzerland	92 (63, 120)[a]	38 (24, 53)[a]	75 (60, 91)[a]	42 (30, 54)[a]	38 (29, 47)[a]	.
2	Germany	26 (10, 41)[a]	68 (50, 87)[a]	109 (87, 131)[a]	43 (30, 57)[a]	-7 (-22, 9)	3 (-3, 8)
2	Finland	7 (-4, 17)	36 (22, 50)[a]	125 (103, 148)[a]	81 (61, 100)[a]	12 (2, 23)[b]	.
2	France	68 (46, 89)[a]	49 (39, 60)[a]	62 (48, 76)[a]	3 (-4, 10)	-3 (-9, 4)	1 (-2, 5)
2	Netherlands	72 (51, 94)[a]	82 (63, 101)[a]	62 (49, 75)[a]	48 (38, 57)[a]	48 (39, 57)[a]	.
2	Sweden	73 (52, 93)[a]	17 (7, 28)[a]	39 (28, 50)[a]	34 (22, 46)[a]	3 (-5, 12)	.
3	Austria	92 (68, 117)[a]	104 (85, 123)[a]	127 (104, 150)[a]	80 (63, 96)[a]	49 (34, 64)[a]	.
3	Belgium	153 (113, 193)[a]	37 (24, 50)[a]	71 (55, 87)[a]	25 (16, 35)[a]	27 (16, 37)[a]	.
3	Spain	141 (96, 186)[a]	55 (41, 68)[a]	80 (61, 99)[a]	24 (17, 31)[a]	35 (23, 46)[a]	.
3	England and Wales	117 (82, 151)[a]	82 (59, 104)[a]	59 (45, 73)[a]	60 (46, 74)[a]	26 (16, 35)[a]	.
3	Northern Ireland	85 (60, 109)[a]	86 (63, 109)[a]	63 (42, 84)[a]	65 (43, 87)[a]	93 (71, 114)[a]	.
3	Scotland	102 (71, 133)[a]	97 (77, 117)[a]	76 (57, 95)[a]	78 (57, 99)[a]	41 (26, 56)[a]	.
3	Portugal	87 (65, 109)[a]	97 (61, 133)[a]	89 (69, 110)[a]	21 (9, 33)[a]	9 (-7, 25)	.
4	Czechia	151 (111, 191)[a]	269 (224, 315)[a]	94 (76, 112)[a]	31 (19, 43)[a]	33 (20, 45)[a]	.
4	Estonia	18 (-2, 38)[c]	221 (185, 256)[a]	119 (95, 143)[a]	11 (-13, 35)	-5 (-27, 17)	1 (-4, 7)
4	Greece	56 (40, 72)[a]	170 (141, 200)[a]	128 (104, 152)[a]	-2 (-13, 9)	-36 (-52, -19)[a]	11 (5, 16)[a]
4	Hungary	100 (66, 134)[a]	270 (228, 313)[a]	84 (69, 100)[a]	9 (-4, 22)	4 (-11, 19)	.
4	Italy	169 (129, 209)[a]	120 (101, 138)[a]	132 (110, 154)[a]	43 (33, 52)[a]	16 (4, 28)[a]	.
4	Slovenia	143 (100, 187)[a]	107 (80, 133)[a]	67 (49, 86)[a]	13 (-5, 30)	-4 (-20, 12)	1 (-3, 5)
4	United States of America (USA)	136 (117, 154)[a]	160 (142, 178)[a]	90 (77, 102)[a]	16 (11, 20)[a]	-9 (-14, -3)[a]	2 (1, 3)[a]
5	Bulgaria	219 (154, 283)[a]	622 (533, 711)[a]	225 (178, 272)[a]	6 (-7, 20)	34 (15, 52)[a]	.
5	Croatia	126 (89, 164)[a]	298 (252, 344)[a]	170 (141, 200)[a]	42 (27, 57)[a]	53 (34, 72)[a]	.
5	Lithuania	193 (151, 234)[a]	386 (335, 437)[a]	235 (202, 268)[a]	97 (76, 117)[a]	114 (95, 134)[a]	.
5	Latvia	37 (14, 61)[a]	353 (299, 407)[a]	149 (124, 175)[a]	-5 (-31, 20)	-109 (-138, -81)[a]	21 (14, 28)[a]
5	Poland	160 (119, 202)[a]	279 (236, 323)[a]	84 (67, 101)[a]	-37 (-46, -29)[a]	-70 (-79, -60)[a]	20 (17, 24)[a]
5	Slovakia	86 (62, 109)[a]	356 (303, 409)[a]	106 (87, 125)[a]	5 (-6, 15)	-5 (-17, 6)	1 (-1, 3)

Note:
a. p<0.01, b. p<0.05, c. p<0.1, d. Excess deaths per 100k people.
e. Harvesting effect ratio was defined as the proportion of cumulative excess mortality in 2020-2022 or 2020-2023 that was offset by cumulative negative excess mortality during 2023 and 2024 combined or 2024 alone;
f. A harvesting percentage exceeding 100% indicates that the subsequent mortality deficit entirely offset or surpassed the initial mortality surge, while missing values suggest that the required pattern of all-positive surge years followed by all-negative deficit years was not observed in that group. Therefore, no harvesting effect was computed.

© 2026 Chen X et al. *JAMA Network Open*.

eTable 7. Annual Excess Death Rate per 100,000 Population and Estimated Harvesting Effect, by Country, 2020-2024, Relative to Expected Deaths Based on 2015-2019 Mortality (the Benjamini-Hochberg FDR Adjustment)

Quintile	Country	2020	2021	2022	2023	2024	Harvesting effect ratio (%)
1	Australia	-16 (-21, -11)[a]	15 (10, 20)[a]	81 (67, 94)[a]	38 (31, 46)[a]	39 (32, 46)[a]	.
1	Denmark	-21 (-31, -10)[a]	12 (-2, 25)[c]	35 (23, 46)[a]	-2 (-13, 9)	-44 (-57, -30)[a]	180 (-217, 577)
1	Iceland	3 (-25, 31)	9 (-18, 37)	99 (64, 133)[a]	58 (29, 87)[a]	60 (28, 92)[a]	.
1	Israel	28 (19, 36)[a]	42 (34, 50)[a]	39 (29, 48)[a]	5 (-10, 20)	14 (9, 19)[a]	.
1	Luxembourg	25 (1, 49)[b]	1 (-20, 22)	-22 (-43, -2)[b]	-45 (-67, -24)[a]	-54 (-75, -33)[a]	2770 (-41884, 47424)
1	Norway	-9 (-18, -0)[b]	14 (1, 27)[b]	77 (62, 92)[a]	35 (24, 45)[a]	35 (24, 46)[a]	.
1	New Zealand	-45 (-56, -35)[a]	-16 (-23, -10)[a]	40 (30, 50)[a]	8 (-0, 17)[c]	-22 (-29, -15)[a]	.
2	Canada	33 (25, 42)[a]	32 (26, 38)[a]	76 (65, 87)[a]	33 (27, 39)[a]	-3 (-11, 5)	2 (-2, 5)
2	Switzerland	94 (62, 125)[a]	41 (28, 54)[a]	79 (62, 95)[a]	46 (34, 58)[a]	43 (32, 53)[a]	.
2	Germany	28 (12, 43)[a]	71 (53, 89)[a]	113 (91, 136)[a]	49 (36, 62)[a]	-0 (-14, 14)	0 (-3, 3)
2	Finland	6 (-4, 16)	36 (23, 49)[a]	125 (104, 146)[a]	80 (61, 100)[a]	12 (1, 23)[b]	.
2	France	73 (51, 96)[a]	57 (44, 69)[a]	71 (55, 88)[a]	14 (6, 22)[a]	11 (3, 18)[a]	.
2	Netherlands	76 (54, 98)[a]	87 (67, 106)[a]	68 (54, 81)[a]	55 (45, 65)[a]	57 (48, 66)[a]	.
2	Sweden	73 (54, 92)[a]	18 (7, 29)[a]	40 (28, 51)[a]	35 (23, 46)[a]	4 (-5, 14)	.
3	Belgium	153 (109, 196)[a]	36 (23, 49)[a]	70 (54, 86)[a]	24 (14, 34)[a]	25 (15, 34)[a]	.
3	Spain	147 (99, 194)[a]	63 (49, 77)[a]	91 (71, 111)[a]	37 (28, 46)[a]	49 (34, 64)[a]	.
3	England and Wales	119 (85, 154)[a]	85 (64, 107)[a]	64 (48, 79)[a]	66 (51, 81)[a]	32 (22, 42)[a]	.
3	Northern Ireland	85 (61, 109)[a]	86 (62, 111)[a]	64 (44, 83)[a]	66 (42, 89)[a]	93 (72, 115)[a]	.
3	Scotland	103 (73, 133)[a]	99 (80, 118)[a]	78 (59, 97)[a]	81 (60, 101)[a]	44 (28, 59)[a]	.
3	Portugal	87 (65, 108)[a]	96 (62, 131)[a]	89 (67, 110)[a]	20 (8, 32)[a]	8 (-8, 23)	.
3	Slovenia	144 (96, 192)[a]	107 (81, 134)[a]	68 (48, 88)[a]	14 (-4, 31)	-2 (-19, 14)	1 (-3, 4)
4	Austria	91 (67, 116)[a]	103 (84, 123)[a]	126 (104, 148)[a]	79 (62, 95)[a]	47 (31, 64)[a]	.
4	Czechia	151 (113, 189)[a]	269 (226, 312)[a]	94 (75, 113)[a]	30 (18, 43)[a]	32 (20, 45)[a]	.
4	Estonia	18 (-3, 40)[c]	221 (187, 255)[a]	119 (95, 143)[a]	11 (-13, 35)	-5 (-28, 18)	1 (-4, 7)
4	Greece	56 (40, 73)[a]	171 (139, 203)[a]	129 (105, 152)[a]	-1 (-12, 11)	-34 (-51, -17)[a]	10 (4, 15)[a]
4	Hungary	100 (65, 135)[a]	271 (228, 313)[a]	85 (69, 102)[a]	10 (-2, 22)[c]	5 (-10, 21)	.
4	Italy	174 (135, 213)[a]	127 (108, 147)[a]	141 (115, 167)[a]	54 (43, 66)[a]	30 (18, 42)[a]	.
4	United States of America (USA)	141 (120, 162)[a]	166 (148, 185)[a]	98 (84, 111)[a]	25 (21, 30)[a]	3 (-2, 7)	.
5	Bulgaria	220 (158, 281)[a]	624 (536, 711)[a]	227 (182, 271)[a]	9 (-5, 22)	37 (18, 55)[a]	.
5	Croatia	128 (90, 166)[a]	300 (249, 350)[a]	173 (142, 203)[a]	45 (29, 61)[a]	56 (37, 75)[a]	.
5	Lithuania	193 (152, 234)[a]	386 (337, 436)[a]	235 (200, 270)[a]	97 (78, 116)[a]	115 (94, 135)[a]	.
5	Latvia	38 (12, 64)[a]	353 (302, 405)[a]	150 (122, 178)[a]	-5 (-30, 20)	-109 (-139, -79)[a]	21 (14, 28)[a]
5	Poland	159 (117, 201)[a]	278 (234, 322)[a]	83 (67, 99)[a]	-39 (-48, -30)[a]	-71 (-81, -62)[a]	21 (17, 25)[a]
5	Slovakia	86 (63, 109)[a]	356 (303, 410)[a]	106 (89, 124)[a]	5 (-7, 17)	-5 (-16, 7)	1 (-1, 3)

Note:
a. q<0.01, b. q<0.05, c. q<0.1, d. Excess deaths per 100k people.
e. Harvesting effect ratio was defined as the proportion of cumulative excess mortality in 2020-2022 or 2020-2023 that was offset by cumulative negative excess mortality during 2023 and 2024 combined or 2024 alone;
f. A harvesting percentage exceeding 100% indicates that the subsequent mortality deficit entirely offset or surpassed the initial mortality surge, while missing values suggest that the required pattern of all-positive surge years followed by all-negative deficit years was not observed in that group. Therefore, no harvesting effect was computed.

© 2026 Chen X et al. *JAMA Network Open*.
