# Supplementary material for: Global Assessment of COVID-19 Mortality Displacement From 2020 to 2024
Source: JAMA Netw Open. 2026 Jan 29;9(1):e2555442. doi: 10.1001/jamanetworkopen.2025.55442 (PMC12856679; doi:10.1001/jamanetworkopen.2025.55442)
Supplement: Supplement 2. — Data Sharing Statement [file jamanetwopen-e2555442-s002.pdf]

Data Sharing Statement

Chen. Global Assessment of COVID-19 Mortality Displacement From 2020 to 2024. *JAMA Netw Open*. Published January 29, 2026. doi:10.1001/jamanetworkopen.2025.55442

Data
Data available: No

Additional Information
Explanation for why data not available: Original datasets from the Short-term Mortality Fluctuations (STMF) data series of the Human Mortality Database are publicly available.
